# Nitric Oxide Overproduction in Tomato *shr* Mutant Shifts Metabolic Profiles and Suppresses Fruit Growth and Ripening

**DOI:** 10.3389/fpls.2016.01714

**Published:** 2016-11-28

**Authors:** Reddaiah Bodanapu, Suresh K. Gupta, Pinjari O. Basha, Kannabiran Sakthivel, Sadhana, Yellamaraju Sreelakshmi, Rameshwar Sharma

**Affiliations:** Repository of Tomato Genomics Resources, Department of Plant Sciences, School of Life Sciences, University of HyderabadHyderabad, India

**Keywords:** tomato, nitric oxide, fruit ripening, metabolites, molecular mapping

## Abstract

Nitric oxide (NO) plays a pivotal role in growth and disease resistance in plants. It also acts as a secondary messenger in signaling pathways for several plant hormones. Despite its clear role in regulating plant development, its role in fruit development is not known. In an earlier study, we described a *short root* (*shr*) mutant of tomato, whose phenotype results from hyperaccumulation of NO. The molecular mapping localized *shr* locus in 2.5 Mb region of chromosome 9. The *shr* mutant showed sluggish growth, with smaller leaves, flowers and was less fertile than wild type. The *shr* mutant also showed reduced fruit size and slower ripening of the fruits post-mature green stage to the red ripe stage. Comparison of the metabolite profiles of *shr* fruits with wild-type fruits during ripening revealed a significant shift in the patterns. In *shr* fruits intermediates of the tricarboxylic acid (TCA) cycle were differentially regulated than WT indicating NO affected the regulation of TCA cycle. The accumulation of several amino acids, particularly tyrosine, was higher, whereas most fatty acids were downregulated in *shr* fruits. Among the plant hormones at one or more stages of ripening, ethylene, Indole-3-acetic acid and Indole-3-butyric acid increased in *shr*, whereas abscisic acid declined. Our analyses indicate that the retardation of fruit growth and ripening in *shr* mutant likely results from the influence of NO on central carbon metabolism and endogenous phytohormones levels.

## Introduction

Nitric oxide (NO) is a bioactive gaseous molecule that participates in a plethora of plant development responses right from seed germination to plant senescence. It acts as a multifunctional signaling molecule regulating a range of developmental processes in conjunction with almost all major phytohormones (Freschi, 2013). Several evidences have indicated that the interplay between auxin and NO regulates cucumber adventitious roots development (Pagnussat et al., 2003), tomato lateral root formation (Correa-Aragunde et al., 2004). Similarly, cytokinin (CK) and NO synergistically and antagonistically regulate several developmental processes of plants (Liu et al., 2013). It is reported that NO and gibberellic acid (GA) interact in seed germination (Bethke et al., 2007) and hypocotyl growth during de-etiolation process (Lozano-Juste and León, 2011), wherein NO acts upstream to GA. During seed germination, NO appears to negate abscisic acid (ABA) effects and enhance germination by activation of transcription of ABA catabolism gene CYP707A2 and NO sensing gene ERFVII (Liu et al., 2009; Gibbs et al., 2014). On the contrary, NO also participates in many ABA signaling events particularly G protein-coupled signaling cascades (Wang et al., 2001).

During recent years, many studies reported that cold stress can increase the production of NO in seeds (Bai et al., 2012), leaves (Zhao et al., 2009; Cantrel et al., 2011) and fruits (Xu et al., 2012). Considering that NO signaling operates during cold stress, the fumigation of fruits with NO gas has been used to prevent chilling injury during cold storage (Singh et al., 2009; Zaharah and Singh, 2011). Studies on NO fumigation to fruits also indicated its involvement in the ripening of both climacteric and non-climacteric fruits. The prevention of chilling injury by NO has been attributed to several factors including delay in climacteric phase by antagonizing ethylene synthesis (Manjunatha et al., 2012), protecting fruits from pathogens and impeding ripening and/or senescence (Singh et al., 2013). The NO treatment delayed the ripening by suppressed respiration rate, reduced ethylene biosynthesis and chilling injury, delayed development of browning disorders, disease incidence, and skin color changes, flesh softening and reduced activity of softening enzymes (Leshem and Pinchasov, 2000; Manjunatha et al., 2010).

Currently information about the influence of NO on molecular processes regulating fruit ripening is largely restricted to post-harvest fruits stored in cold (Manjunatha et al., 2014). NO fumigation of cold-stored mango fruits increased the levels of tartaric acid and shikimic acids (Zaharah and Singh, 2011). NO treatment of peach fruits increased palmitoleic, oleic, and linolenic acids, while decreased linoleic acid levels (Zhu and Zhou, 2006). The softening of banana fruits was retarded by NO by lowering the activity of cell wall degrading enzymes pectin methylesterase (PME) and β-1-4-endoglucanase (Cheng et al., 2009). In peach and kiwi fruits NO upregulated the activity of enzymes involved in quenching of reactive oxygen species such as catalase, peroxidases and superoxide dismutase (SOD) (Flores et al., 2008; Zhu et al., 2008). Exogenous NO delayed tomato ripening via transcriptional suppression of ethylene biosynthesis genes ACC synthase (*ACS*) and ACC oxidase (*ACO*) (Eum et al., 2009). In pepper fruits, ripening is associated with an increase in the nitration of proteins and exogenous treatment of NO delayed ripening by blocking protein nitration (Chaki et al., 2015).

Most studies examining the role of NO in plant development including fruit ripening are largely confined to exogenous application of NO and its agonists and antagonists. This is related to the dearth of mutants affected in NO levels in the higher plants. The paucity of mutants may be related to the multiplicity of pathways for NO generation in plants depending on the tissue and ambient conditions (Gupta et al., 2011). Characterization of Arabidopsis NO mutants revealed that the *in-vivo* level of NO is reportedly regulated by mutations in diverse genes. The mutation in the *cGTPase* gene in *nos1/noa1* mutant (Guo et al., 2003; Moreau et al., 2008) lowered NO levels and stimulated early flowering. In contrast mutation in *CUE1* gene encoding a chloroplast phosphoenolpyruvate/phosphate translocator enhanced NO levels and delayed flowering (He et al., 2004). The null alleles of *HOT5* locus encoding S-nitrosoglutathione reductase (GSNOR) display decreased tolerance to temperature stress associated with increase in levels of nitrate, NO and nitroso species (Lee et al., 2008). An increase in NO level in arginase negative mutants stimulated lateral roots while reduction in NO level in prohibitin (*PHB3*) gene mutant reduced auxin-induced lateral root formation (Wang et al., 2010).

Considering that exogenous NO influences post-harvest fruit ripening it would be of interest to examine how endogenous NO regulates fruit ripening and associated cellular metabolism. In this study, we compared fruit ripening in the *sh*ort *r*oot (*shr*) mutant of tomato that hyperaccumulates NO (Negi et al., 2010, 2011) with its wild type (WT) progenitor. We report that *shr* mutation prominently affects the fruit growth and delays ripening probably through its effect on cellular homeostasis. Profiling of plant hormones in *shr* and wild-type fruits revealed changes in accumulation patterns of ABA, indole-3-acetic acid (IAA) and indole-3-butyric acid (IBA) that may have influenced the observed metabolic shifts. We also mapped *shr* locus on chromosome nine of tomato. However, its identity remained elusive.

## Materials and methods

### Plant materials

The *shr* mutant of *Solanum lycopersicum* cv Ailsa Craig (wild type- WT) was isolated from a γ-irradiated M_2_ population of tomato as described in Negi et al. (2010). *S. pennellii* [LA 716] and *S. pimpinellifolium* [LA1589] (SP) seeds were obtained from Tomato Genetics Resource Center (UC, Davis, USA). The plants were grown in the greenhouse at Hyderabad under natural photoperiod (12–14 h day, 10–12 h night) at 28 ± 1°C during the day and ambient temperature (14–18°C) in the night. The RH in the greenhouse ranged from 45–70%.

A F_2_ mapping population consisting of 69 plants was generated, segregating for short root phenotype, from an interspecific F_1_ hybrid (*S. lycopersicum shr/shr* x *S. pennellii SHR*/*SHR*). Owing to self-incompatibility, the F_1_ plants were selfed by manual sib mating. A second F_2_ mapping population was generated which consist of 769 plants, segregating for short root phenotype, from an interspecific F_1_ hybrid (*S. lycopersicum shr/shr* x *S. pimpinellifolium SHR*/*SHR*). The F_2_ seedlings were scored for root length and NO levels as described in Negi et al. (2010). For NO determination detached roots were submerged in 10 μM of DAF-2 DA fluorescent probe in 10 mM MES-KCl (pH 7.0) buffer for 20 min. Thereafter the roots were washed with 10 mM MES-KCl (pH 7.0) buffer for 15 min. The NO level was examined by epi-fluorescence using the U-MWIB2 mirror unit (excitation 495 nm, emission 515 nm) in the Olympus BX-51 Microscope (Negi et al., 2010).

After scoring the seedling phenotypes, seedlings were transferred to the pots and plants were grown in the greenhouse. Wherever possible, F_3_ seedlings were used to confirm the phenotype of F_2_ plants. Chi-square tests were performed to determine the goodness of fit between the Mendelian ratio of the F_2_ mapping population and the segregation data for the short root (*shr*) and the molecular markers.

### Estimation of ethylene, pigments, brix and fruit firmness

For estimation of ethylene, fruits were harvested at different ripening stages viz., mature green (MG), breaker (BR) and red ripe (RR) stage. The ethylene emission from the harvested fruits was measured using a previously described procedure (Kilambi et al., 2013). Chlorophylls and carotenoids were extracted from leaves from 7–8 internodes of 45-day-old plants in 80% (v/v) acetone using the protocol of Makeen et al. (2007) and their amounts were calculated using the equation of Lichtenthaler (1987). Carotenoids were extracted from the pericarp of MG, BR and RR fruits using the procedure of Gupta et al. (2015). To avoid photooxidation, the entire procedure was performed under dim light. The carotenoids amount from the fruit tissue was calculated by comparing the peak area with the peak area obtained using pure standards of each carotenoid. For determination of sugars, the entire pericarp of fruit was homogenized, and values were recorded using PAL-1 refractometer. The firmness of fruits was measured three times at equatorial plane using Durofel DFT 100 (Gupta et al., 2014).

### Determination of endogenous no levels in fruits

The endogenous levels of NO at MG and RR stage of fruits was determined by using EPR spectroscopy, and also the fruits cells were examined for DAF-2 DA fluorescence following the protocols described in Negi et al. (2010). However, both methods could not detect the NO indicating that NO level in fruits was below the limit of detection.

### Extraction of primary metabolites and GC-MS data processing

The metabolite profiling of fruits of WT and *shr* was essentially carried out by following the protocol of Roessner et al. (2000). The fruits from MG, BR, and RR stage were ground to a fine powder in liquid nitrogen. A 100 mg fresh weight of fruit powder was mixed with 1.4 mL 100% methanol and 60 μL of internal standard ribitol (0.2 mg/ml, w/v). After mixing, the sample was shaken at 70°C in a thermomixer for 15 min at 950 rpm. After that, 1.4 mL MilliQ water was added and after thorough mixing the sample was transferred in GL-14 Schott Duran glass vial and centrifuged at 2200 g for 15 min. An aliquot of polar phase (150 μL) was transferred in fresh Eppendorf tube and dried by vacuum centrifugation for 3–4 h. The dried sample was derivatized; first, it was dissolved in 80 μL of methoxyamine hydrochloride (20 mg/mL) and incubated at 37°C for 90 min at 600 rpm. Thereafter, 80 μL of MSTFA was added, and incubation was carried out at 37°C for 30 min at 600 rpm. The derivatized sample was transferred to a GC-MS injection vial and analyzed by Leco-PEGASUS GCXGC-TOF-MS system (Leco Corporation, USA) equipped with 30 m Rxi-5 ms column with 0.25 mm i.d. and 0.25 μm film thickness (Restek, USA). The injection temperature, interface, and ion source were set at 230°, 250°, and 200°C respectively. For the proper separation of groups of metabolites, the run program was set as following; isothermal heating at 70°C for 5 min, followed by 5°C min^−1^ oven temperature ramp to 290°C and then final heating at 290°C for 5 min. The carrier gas (helium gas) flow rate was set to 1.5 mL/min. A 1 μL of sample was injected in split less mode and mass spectra were recorded at 2 scans/sec within a mass-to-charge ratio range 70–600.

The raw data were processed by ChromaTOF software 2.0 (Leco Corporation, USA) and further analyzed using the MetAlign software package (Lommen and Kools, 2012; www.metalign.nl) with a signal to noise ratio of ≥ 2, for base line correction, noise estimation, alignment and extraction of ion-wise mass signal. The mass signals that were present in less than three samples were discarded. The Metalign results were processed with MSClust software for reduction of data and compound mass extraction (Tikunov et al., 2012). The mass spectra extracted by MSClust were opened in NIST MS Search v 2.2 software for the identification of compound name within the NIST (National Institute of Standard and Technology) Library, and Golm Metabolome Database Library. The compound hits which showed maximum matching factor (MF) value (>600) and least deviation from the retention index (RI) was used for metabolite identity. The data was analyzed by normalizing with the internal standard ribitol.

### Extraction of phytohormones and LC-MS analysis

The phytohormone extraction from the fruit sample of WT and *shr* was performed as described in Pan et al. (2004). A 100 mg homogenized powder from fruit sample was mixed with 500 μL of pre-chilled extraction solvent consisting of 2-propanol: MilliQ water: concentrated HCL in the ratio of 2:1:0.002 (v/v/v) respectively. After mixing, the extraction was carried out by shaking the sample for 30 min at 4°C at 500 rpm. Thereafter, 1 mL of dichloromethane (DCM) was added to the sample mix, and the incubation was continued for 30 min at 4°C at 500 rpm. After centrifugation at 13,000 g for 15 min at 4°C, the supernatant (~ 900 μL) was transferred to a fresh Eppendorf tubes and dried completely using the Speedvac (Thermo Scientific, USA). Before injection, the dried residue was dissolved in 70 μL of precooled 100% methanol followed by centrifugation at 13,000 g for 5 min.

The sample was transferred to an injection vial and analyzed using UPLC/ESI-MS (Waters, Milford, MA USA). The system consists of an Aquity UPLC™ System, quaternary pump, and autosampler. For separation of hormones, the sample was analyzed on a Hypercil GOLD C_18_ (Thermo Scientific) column (2.1 × 75 mm, 2.7 μm). A gradient elution program was performed using two solvents system, solvent A- containing ultrapure water with 0.1% (v/v) formic acid, solvent B- containing acetonitrile with 0.1% (v/v) formic acid and run for 9 min at 20°C. The abscisic acid (ABA), jasmonic acid (JA), and salicylic acid (SA) detection was performed on Exactive^*TM*^Plus Orbitrap mass spectrometer (Thermo Fisher Scientific, USA) in all ion fragmentation (AIF) mode (range of m/z 50–450) equipped with heated electrospray ionization (ESI) in negative ion mode. The zeatin, IAA, IBA, epibrassinosteroids (Epi-BR) and methyl jasmonate (MeJA) were analyzed in positive ion mode. For both modes, the following instruments setting were used, capillary temperature −350°C, sheath gas flow (N_2_) 35 (arbitrary units), AUX gas flow rate (N_2_) 10 (arbitrary units), collision gas (N_2_) 4 (arbitrary units) and the capillary voltage 4.5 kV under ultra-high vacuum 4e^−10^ mbar. The hormones were analyzed from the >5 different fruits harvested from ripening stages viz. MG, BR, and RR of WT and *shr*. The quantification of each hormone was carried out by comparing the peak areas with those obtained for the respective hormone standards.

### Principal component analysis (PCA)

To obtain the overall clustering of the samples, we performed PCA using Metaboanalyst 3.0 (Xia et al., 2015; http://www.metaboanalyst.ca/). Firstly, we took the average of metabolites from at least three replicates and analyzed the differences in metabolites accumulation across the ripening stages in both WT and *shr* and then the results were presented in a two-dimensional graphical display.

### Construction of primary metabolite pathways

All the metabolites measured using the GC-MS methods were mapped to the general metabolic pathways as described in the KEGG (Kyoto Encyclopedia of Genes and Genomes, http://www.genome.jp/kegg) and LycoCyc (Sol Genomic networks, http://solcyc.solgenomics.net/). To compare levels of each metabolite across the ripening stages (MG, BR, and RR), we performed all pairwise multiple comparison procedures (Student-Newman-Keuls Method) by One-Way ANOVA using Sigma Plot version 11 with a significance threshold *P* ≤ 0.05 to highlight patterns of change across the ripening stages in *shr* compared to WT. Average fold change of metabolites occurring across the ripening stages in *shr* fruits compared to WT was shown on a primary metabolite pathway as presented in Do et al. (2010). A log2 fold of 0 means no difference, a log2-fold of 0.5 means 1or higher fold changes (equal to average means 1.5-fold) 1 means 2-fold or higher, a log2-fold of two means 4-fold or higher, and so on.

### Metabolites and hormones correlation networks creation

Networks for both WT and *shr* were created using Cytoscape software package (http://www.cytoscape.org/; Cline et al., 2007). Nodes represent the metabolites (circle), hormones (hexagon shape) and edges represent connectivity between the two metabolites. The connectivity between two nodes is drawn if the Pearson's Correlation Coefficient (PCC) value is larger than 0.9 either in positive or negative mode. New correlations in the *shr* network, which were insignificant in WT at all ripening stages were considered as new associations in *shr* or vice-versa.

### DNA extraction

Genomic DNA was isolated from young leaves (80–100 mg/well) in 96 well deepwell plates using a DNA extraction protocol developed for tomato (Sreelakshmi et al., 2010). The DNA was quantified using Nanodrop (ND-1000) spectrophotometer and DNA samples were diluted to final concentration of 5 ng/μL.

### Screening for polymorphic markers

For mapping *shr* locus, we selected 129 SSR and 6 InDel markers that were evenly distributed across twelve chromosomes of the tomato. These markers were selected from the Solanaceae Genome Network (http://solgenomics.net/) and Tomato Mapping Resource Database (http://www.tomatomap.net/, Last accessed in 2013) and chosen based on their polymorphisms between tomato cultivar Ailsa Craig, S. *pimpinellifolium* and *S. pennellii* (Supplementary Table 1, for marker details). First, the location of mutation was determined to chromosome nine. Thereafter chromosome 9 was genotyped with 24 SSR and 7 CAPS markers. PCR amplification was in total volume of 20 μL containing 20 ng of genomic DNA, 1× PCR buffer (10 mM Tris, 5 mM KCl, 1.5 mM MgCl_2_, 0.1% (w/v) gelatin, 0.005% (v/v) Tween-20, 0.005% (v/v) Np-40, pH 8.8, 0.2 mM dNTPs, 1 μL Taq polymerase and 5 pmoles each of forward and reverse primers. The cycling conditions for amplification were 94°C-5 min, followed by 35 cycles of 94°C-30 s, 57°C-30 s, 72°C-1 min, finally an extension step 72°C-8 min, and held at 4°C. The PCR products were size separated on 3.5% (w/v) agarose gels and gel images were collected with Alpha Imager™ gel documentation system.

### Bulk segregant analysis (BSA) and genotyping

Selected individuals of F_2_ mapping populations of *shr* x *S. pimpinellifolium* and *shr* x *S. pennellii* were screened for polymorphism between bulks. DNA from fifteen *shr* and fifteen long root plants from *shr* x *S. pimpinellifolium* population, and twelve *shr* and twelve long root plants from *shr* x *S. pennellii* population were selected for bulks preparation. Short root and long root DNA bulks were prepared by pooling equivalent amount of DNA from each plant with specific phenotypic segregant of the F_2_ mapping population. The parent lines and the bulks DNA were then subjected to BSA analysis for the identification of the tightly linked marker (Michelmore et al., 1991). Markers which corresponded to short root bulk and short root mutant and differed in the size of the PCR product with both long root parents (*S. pennellii* and *S. pimpinellifolium*) and long root bulks were considered to co-segregate with *shr* phenotype. Markers that were specific between bulks were assessed on debulks along with their parents.

### Screening of additional markers

For saturation of the *shr* locus, 14 SSR markers from Kazusa DNA Research Institute (http://www.kazusa.or.jp/tomato/, Shirasawa et al., 2010a,b),10 SSR markers from Veg Marks a DNA marker database for vegetables (http://vegmarks.nivot.affrc.go.jp, Last accessed in 2013) and 7 CAPS markers viz., At3g63190, C2_At4g02580, C2_At2g29210, C2_At4g02680, C2_At1g02910, C2_At4g03200, and U228448 (http://solgenomics.net) that were specific to chromosome 9 were selected and screened for polymorphism between the parental lines of mapping population (Supplementary Tables 2–4 for marker details). For amplification of CAPS region, 30 ng genomic DNA, 1 μL of 5 pM/ μL primer, 1X PCR buffer (10 mM Tris, 5 mM KCl, 1.5 mM MgCl_2_, 0.1% (w/v) gelatin, 0.005% (v/v) Tween-20, 0.005% (v/v) Np-40, pH 8.8, 0.2 mM dNTPs and 1 μL Taq polymerase were used. After confirming PCR amplification for CAPS locus by agarose gel electrophoresis, the PCR amplicons of the CAPS markers were digested using *Apo*I, *Hinf* I, *Dra*I and *Msp*I (Fermentas) enzymes. Digestion reactions performed according to the supplier's manual and the products were separated on 3.5% (w/v) agarose gels.

### Map construction and linkage analysis

Markers that showed bulk specific segregation along with *shr* phenotype were used for molecular mapping of the *shr* locus. Given the availability of the higher number of F_2_ segregating progeny, we selected *shr* x *S. pimpinellifolium* mapping population for map construction for *shr* locus. Four SSR markers and one CAPS marker were chosen for molecular mapping of the *shr* locus. Total 769 F_2_ plants of *shr* x *S. pimpinellifolium* were genotyped and analyzed by Chi-square test. Map construction was carried out using the MAPMAKER/EXE V.3.0 (Lander et al., 1987; Lincoln and Lander, 1992) program following Kosambi Function (Kosambi, 1943). Linkage groups were determined using “group” and “error detection on” commands with a LOD score of 3.0 and a recombination fraction of 0.5. The “compare” and “order” commands in Mapmaker were used to identify the most probable marker order within a linkage group. The “ripple” command was used to verify and confirm marker order as determined by multipoint analysis. Recombination frequencies were converted into map distances centi-Morgans (cM) using the Kosambi mapping function (Kosambi, 1943), and the linkage group maps were drawn using the MapChartv. 2.1 software (Voorrips, 2002).

### Genome analysis and candidate gene prediction

The tomato genome, ITAG version 2.3 (SGN: http://solgenomics.net/gb2/gbrowse/ITAG2.3_genomic/) was used for overlaying the closest markers encompassing the *shr* locus. The predicted genes in the region encompassing *shr* locus were searched. The information on expression of the predicted genes was found by BLASTN searching of Tomato Expression Database (http://solgenomics.net/ted).

## Results

### Inheritance of *shr* locus

**Figure 1:**
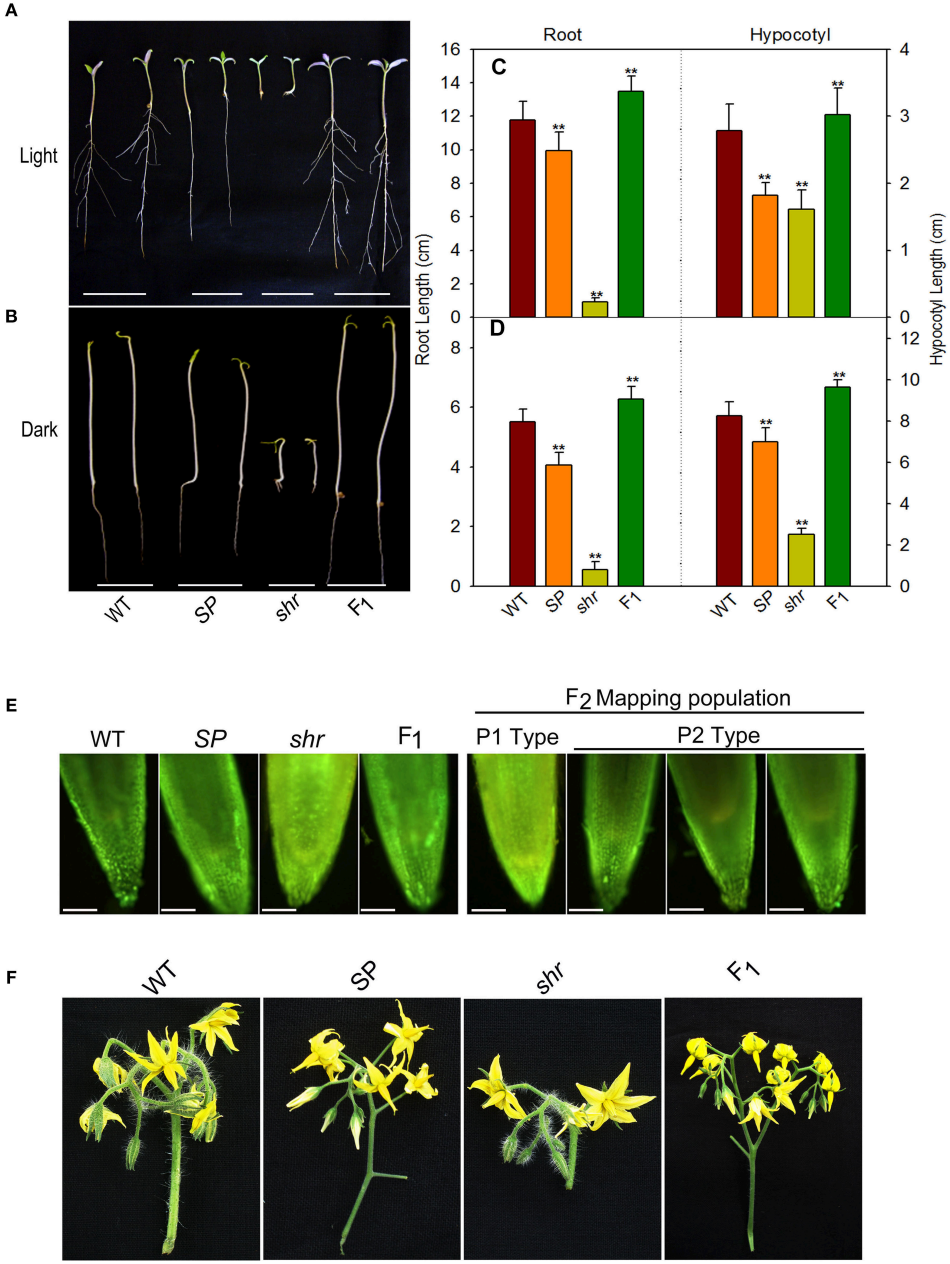
**Genetic segregation of phenotypic traits in ***shr*** mutant**. Seedling phenotype **(A,B)**, root and hypocotyl length **(C,D)** of Ailsa Craig (WT), *S. pimpinellifolium* (*SP*), *short root* (*shr*) mutant and F_1_seedlings grown in light (**A,C-** 9-day old seedlings) and darkness (**B,D-** 5-day old seedlings). **(E)** NO levels in root tips of 9-day old light-grown seedlings of parent plants (*left panel*) and mapping population (*right panel*) using NO-sensitive dye DAF-2DA. In F_2_ mapping population, short-root seedlings showed NO staining similar to *shr* mutant parent(P1 type) and long-root seedlings showed NO staining similar to *SP* parent (P2 type). **(F)** Inflorescence morphology of WT, *SP, shr* and F_1_ plants. The values are the mean ±SD (*n* = 79 seedlings). Asterisk indicates statistically significant difference between WT and *SP, shr*, and F_1_(One-Way ANOVA;^**^ *P* <0.001). In fluorescence microscopic picture of the root, scale bar corresponds to 10x zoom micro scale, Olympus BX51.

We crossed *shr* mutant with *S. pimpinellifolium*, a red fruited wild relative of tomato for mapping of the *shr* gene, and also compared the phenotypes of *shr* mutant plants and the parental lines at the different stages of development. Both light and dark grown seedlings of *shr* mutant showed extremely short roots compared to parental lines (Figures 1A–D). The F_1_ seedlings of *shr* x *S. pimpinellifolium* grown in both light and dark conditions displayed elongated roots like WT and *S. pimpinellifolium*. Examination of root length of F_2_ segregation mapping populations suggested that *shr* locus is encoded by a monogenic recessive locus (Supplementary Table 5). While seedlings of *S. pimpinellifolium* did not form lateral roots, however, light-grown seedlings of F_1_ cross of *shr* x *S. pimpinellifolium* displayed lateral roots like WT (Figure 1A). The etiolated seedlings of WT lacked lateral roots and consequently etiolated seedlings of F_1_ cross of *shr* x *S. pimpinellifolium* roots did not display lateral roots (Figures 1B,D). Interestingly, the lateral root formation in the F_2_ population of *shr* x *S. pimpinellifolium* showed opposite segregation pattern of 1:3, indicating the presence of a locus in *S. lycopersicum* controlling lateral root initiation independently of *shr* locus (Supplementary Table 6).

The shortening of root in the *shr* mutant is associated with hyperaccumulation of NO; therefore, cosegregation of short root phenotype and accumulation of NO was examined by staining the primary root tip with NO-sensitive fluorophore 4, 5-diaminofluorescein diacetate (DAF-2DA) (Correa-Aragunde et al., 2004). *In vivo* imaging of NO levels in parental lines and *shr* mutant showed stronger fluorescence of DAF-2DA in *shr* mutant root tips, while the level of DAF-2 DA fluorescence in root tips of parental lines was nearly similar. The F_1_ plants of *shr* x *S. pimpinellifolium* showed DAF-2 DA fluorescence level that was similar to parental lines indicating the recessive nature of *shr* locus. The imaging of F_2_ population of *shr* x *S. pimpinellifolium* showed DAF-2 DA fluorescence pattern consistent with above results, showing a 3:1 segregation pattern in root tips. The segregation pattern of NO accumulation as visualized by DAF-2 DA fluorescence in F_2_ mapping population was consistent with *shr* phenotype and indicated the cosegregation of NO hyperaccumulation with the short root locus (Figure 1E).

The F_1_ seedlings of *shr* x *S. pimpinellifolium* were slightly taller and had longer internodes than either WT or *S. pimpinellifolium* (Supplementary Figures 1A,B). Similarly, the leaf of F_1_plant was longer than *shr* mutant and possessed chlorophylls and carotenoids similar to WT and *S. pimpinellifolium* (Supplementary Figures 1C,D). However, F_1_ plants showed an intermediate phenotype than either of its progenitors in the number of flowers and the shape of inflorescence (Figure 1F). On the contrary, the RR fruits of F_1_ hybrid (*shr* x *S. pimpinellifolium*) emitted less ethylene (3.18 ± 0.2120 nL/h/g FW) than *S. pimpinellifolium* (14.59 ± 1.11 nL/h/g FW) and *shr* fruits.

### Mapping of *shr* locus

To map the *shr* gene, the SSR markers described in Tomato Mapping Resource Database (http://www.tomatomap.net/, Last accessed in 2013) and Solanaceae Genome Network (http://solgenomics.net) were screened using the bulk segregation analysis (BSA). Out of 135 markers used, only 69 were polymorphic between the *shr* and *S. pimpinellifolium*. These 69 polymorphic markers were used for BSA of *shr* x *S. pimpinellifolium* populations. Among these, two markers, SSR19, and SSR110 showed polymorphism and mapping results indicated that the *shr* locus was located in a region intervening between SSR19-SSR110 on chromosome 9 of tomato. To develop high-resolution molecular map and saturate the region around the *shr* locus, additional markers for chromosome 9 were selected and analyzed for polymorphism between *shr* mutant and *S. pimpinellifolium* (Ohyama et al., 2009; Shirasawa et al., 2010a). The bulk segregation analysis with TGS0213 and C2_At3g63190 markers showed strong linkage with *shr* locus, and these were used for genotyping of entire mapping population (Supplementary Figures 2A–C).

**Figure 2:**
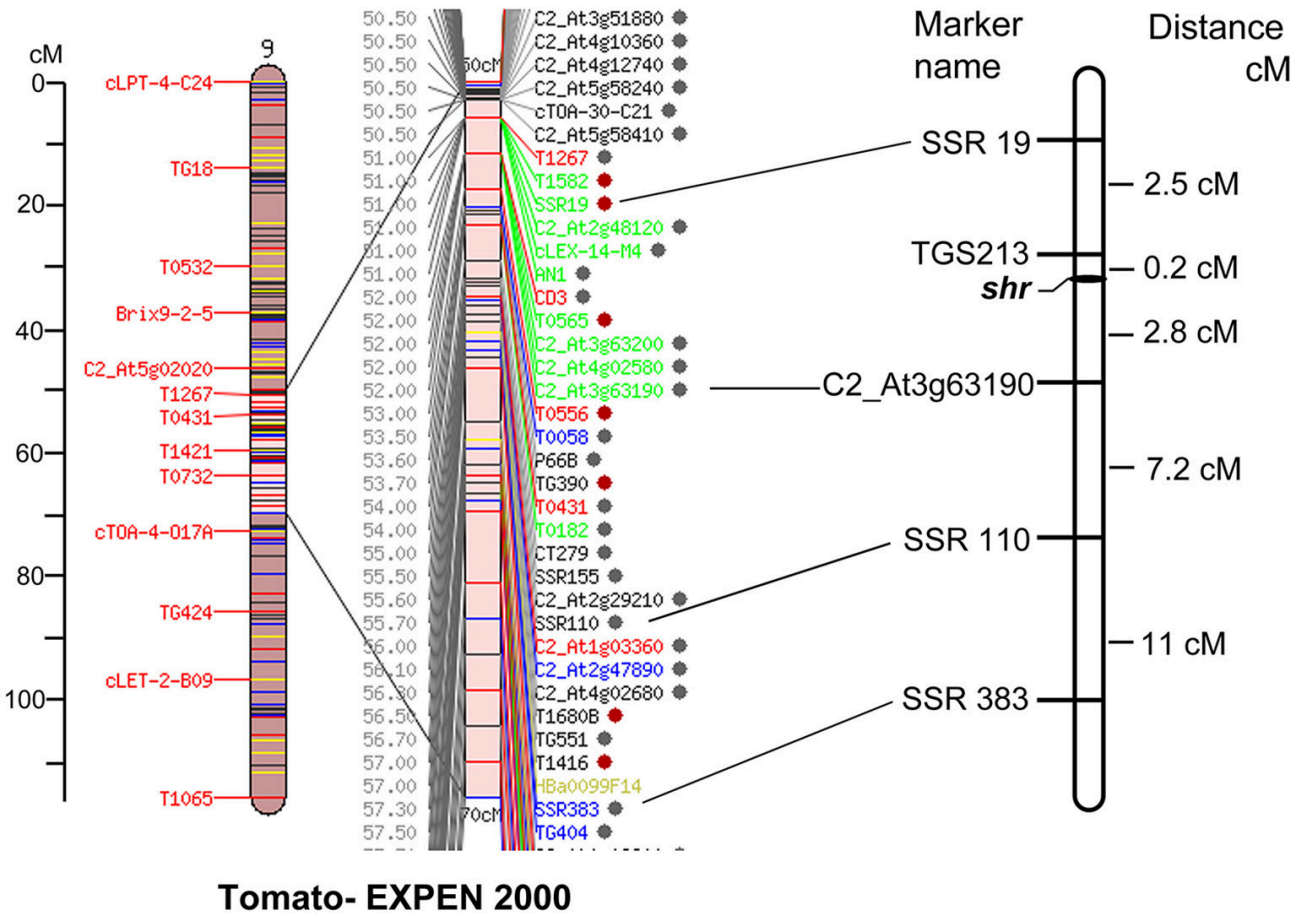
**Genetic map of ***shr*** locus on chromosome 9 using ***shr*** x ***S. pimpinellifolium*** F_**2**_ mapping population (***n*** = 769) (right)**. The Tomato-EXPEN 2000 map (left) shows positions of markers on the map. *shr* locus was identified within TGS0213 and C2_At3g63190 markers. The distances for the maker position are given in centi-Morgan (cM).

A total of six polymorphic markers around *shr* locus, including one CAPS (C2_At3g63190), one InDel (Cosi52) and four SSRs (SSR19, SSR110, SSR383, TGS0213) markers, were genotyped on 769 F_2_*shr* x *S. pimpinellifolium* mapping population. Out of these, five markers showed satisfactorily expected ratio for the co-dominant inheritance of 1:2:1 and were used for mapping the *shr* locus (Supplementary Table 7). Using MAPMAKER3.0 program, the *shr* locus was mapped at 0.2 cM from TGS0213 and 2.8 cM from C2_At3g63190, on chromosome 9 (Figure 2). To identify the candidate gene encoding *shr* locus, the sequence of SSR markers tightly linked to *shr* was searched with BLAST against the tomato genome sequence release ITAG 2.3 Release SL2.40ch09:54045071.58939091(http://solgenomics.net/gb2/gbrowse/ITAG2.3_genomic). The genomic region flanked by two markers was about 4.89 Mb (4894020 bp) and contained 197 genes, which were examined as candidate genes for NO hyperaccumulation. However, the above genomic region is not completely sequenced and consists of two major gaps of size 35116 bp (SL2.40ch09:56426627…56461743) and 31568 bp (SL2.40ch09:56795954…56827522). Currently, it is not known whether these two gaps also harbors functional genes or consist of repetitive DNA sequences. Out of the 197 genes, 139 genes showed high to low expression in tomato root and remaining showed no expression (Supplementary Table 8).

Out of 139 genes showing root specific expression, only three genes were reported to be associated with modulation of cellular NO levels; alcohol dehydrogenase III (ADH3)/GSNO reductase (GSNOR1/HOT5/PAR2, Solyc09g064370 alcohol dehydrogenase III gene), CUE domain containing protein 2 (Solyc09g064860CUE domain containing protein), and glutathione S-transferase (Solyc09g063150 Glutathione S-transferase) (Li et al., 2008; Chen et al., 2009; Lok et al., 2012). The presence of the *shr* mutation in these three genes was examined by amplifying complete ORF of genes from WT and *shr* mutant using PCR and detection of mutation in heteroduplexed DNA using mismatch endonuclease assay (Sreelakshmi et al., 2010). However, no mutation was detected in any of these three genes, thus ruling them out as candidate genes.

### *shr* mutant shows reduced fruit growth and delayed ripening

**Figure 3:**
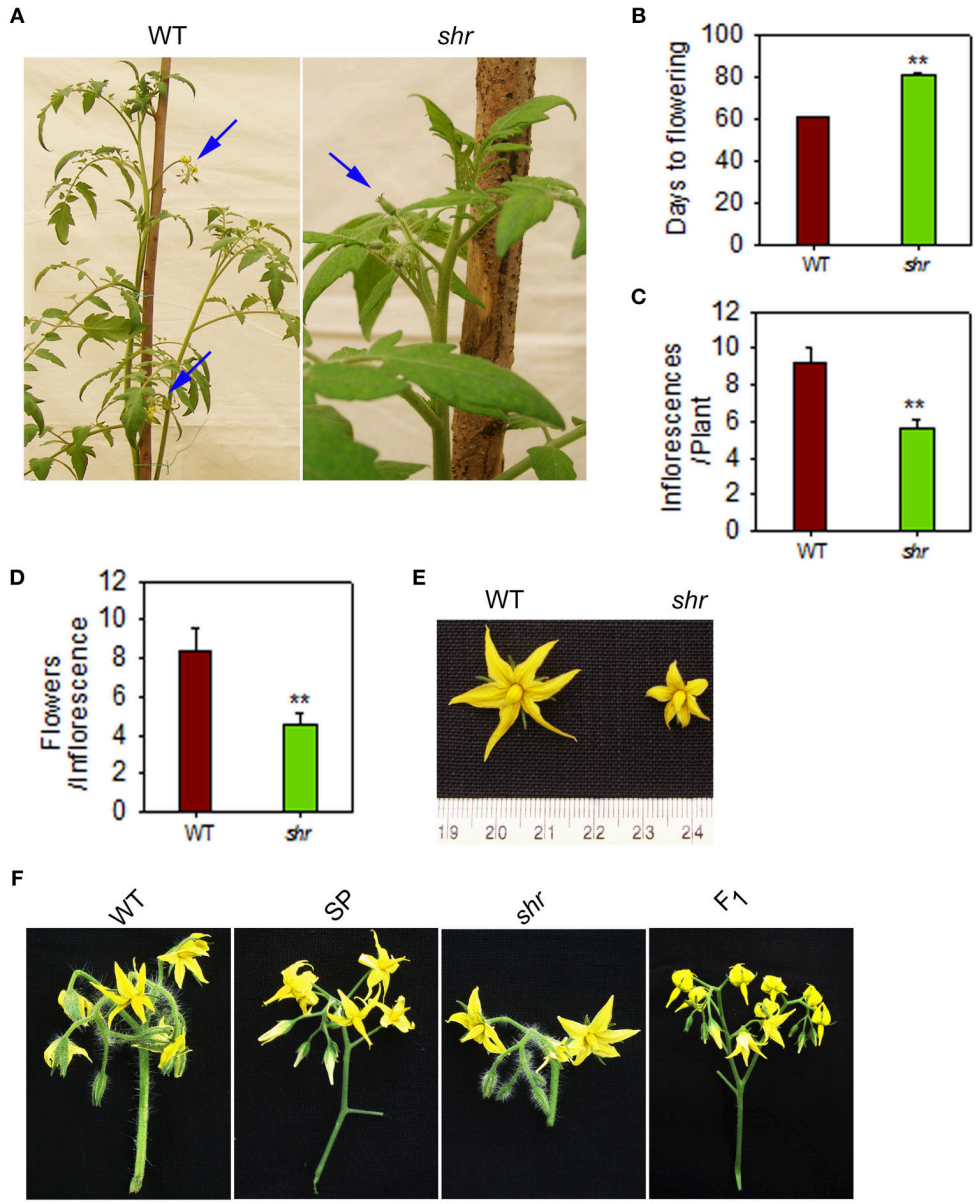
**Inflorescence and floral morphology of ***shr*** mutant plants**. **(A)** Inflorescence(s) in WT and *shr* mutant plants. The blue arrow points that WT plant (60-day old) bears two inflorescences whereas *shr* mutant (80-day old) bear only one inflorescence with unopened flowers. **(B)** Days from sowing for the onset of the flowering. **(C)** Number of inflorescences per plant. **(D)** Number of flowers per inflorescence. **(E)** Floral morphology of WT and *shr* mutant. **(F)** Inflorescence morphology of WT, *S. pimpinellifolium* (SP), *shr* and F1. Note: The peduncle of the inflorescence of *shr* mutant was short and bore less number of flowers compared to WT. The values are the mean ±SD (*n* = 5). Asterisk indicates statistically significant difference between WT and *shr*(One-Way ANOVA; ^**^ *P* < 0.001).

The *shr* mutant shows sluggish growth, prolonged life cycle (*shr*-150 ± 10 days, WT-110 ± 10 days) and a diminutive phenotype with pale green leaves with reduced level of photosynthetic pigments compared to WT (Negi et al., 2010; Supplementary Figure 1D). The pleiotropic effect of *shr* mutation also manifests during reproductive phase. Compared to WT, the initiation of the first inflorescence in the mutant was delayed by nearly 3 weeks (Figures 3A,B). The *shr* mutant made fewer inflorescences with smaller flowers and inflorescence had ca. 50% less flowers than the WT (Figures 3C–F).

**Figure 4:**
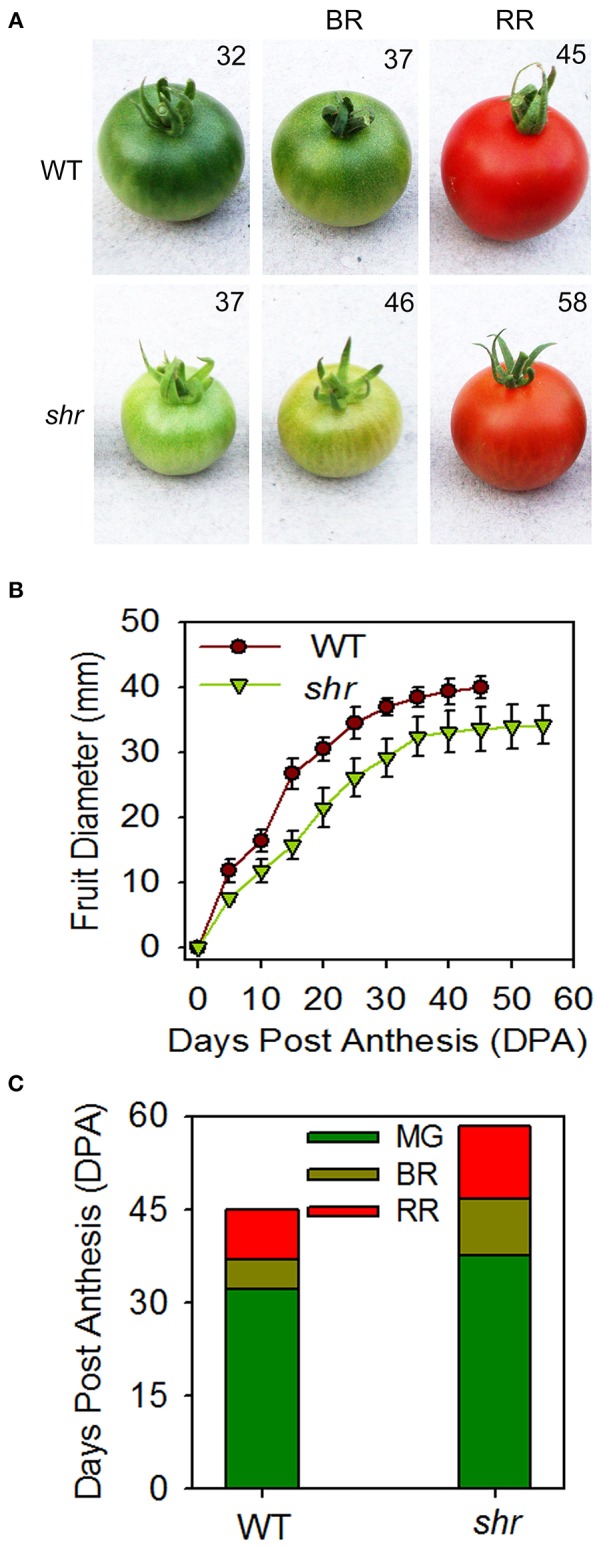
**Fruit development and ripening of ***shr*** fruits. (A)** WT and *shr* fruits harvested at different days from anthesis. **(B)** Time course of fruit development in WT and *shr* mutant from the day of anthesis to RR stage. **(C)** Duration of different ripening phases of WT and *shr* mutant fruits. The values are the mean ±SD (*n* = 5). All the data point statistically significantly different between WT and *shr* were determined throughOne-Way ANOVA (*P* < 0.001).

The influence of *shr* mutation was examined on the chronological development of fruit from anthesis (days post anthesis- DPA) to RR stage. Analogous to delayed inflorescence initiation, the fruit development was slower, and the ripened *shr* fruits were smaller in size than WT (Figures 4A,B). In addition, the *shr* mutation influenced the transition phases of ripening. The time period to reach the MG stage was longer in *shr* fruits (35–37 days *shr*, 30–33 days WT) (Figure 4C). The transition from MG to RR Stage was 7–8 days slower in *shr* fruits than WT. Though *shr* fruits were smaller in size at RR stage, they emitted nearly two-fold higher ethylene than WT (**Figure 7**). Among *shr* and WT fruits, no obvious difference was found in TSS level (Brix) except at RR stage (Supplementary Figure 3A). During ripening, the loss of firmness in *shr* fruits was similar to WT (Supplementary Figure 3B). The pH of WT and *shr* fruits was almost similar (Supplementary Figure 3C). Unlike reduced photosynthetic pigments in *shr* leaves, the accumulation of lycopene and β-carotene in *shr* fruits was only mildly affected. However, the level of carotenoids precursors, phytoene and phytofluene were higher in *shr* fruits than WT (Supplementary Table 9).

### *shr* mutation alters the cellular metabolism during the fruit ripening

**Figure 5:**
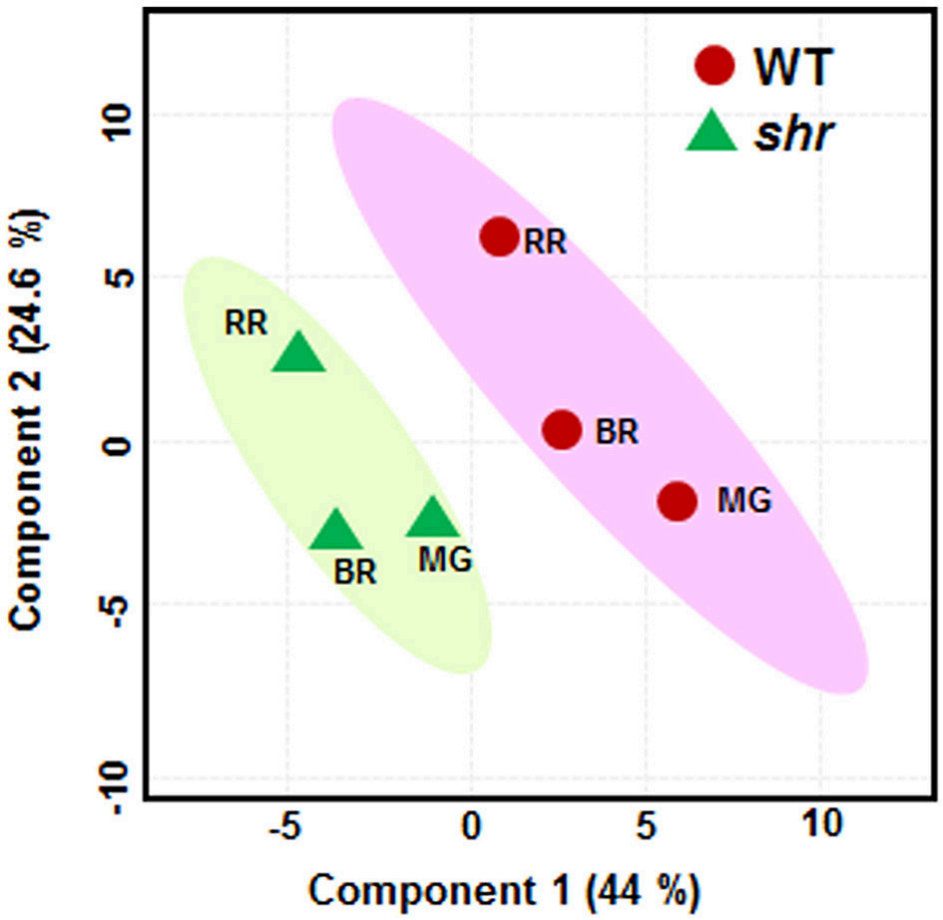
**Principle component analysis (PCA) of metabolic profiles**. The WT and *shr* mutant fruits were analyzed at MG, BR, and RR stage The PCA was constructed using the MetaboAnalyst 3.0. The variance of PC1 and PC2 component is given within parentheses.

**Figure 6:**
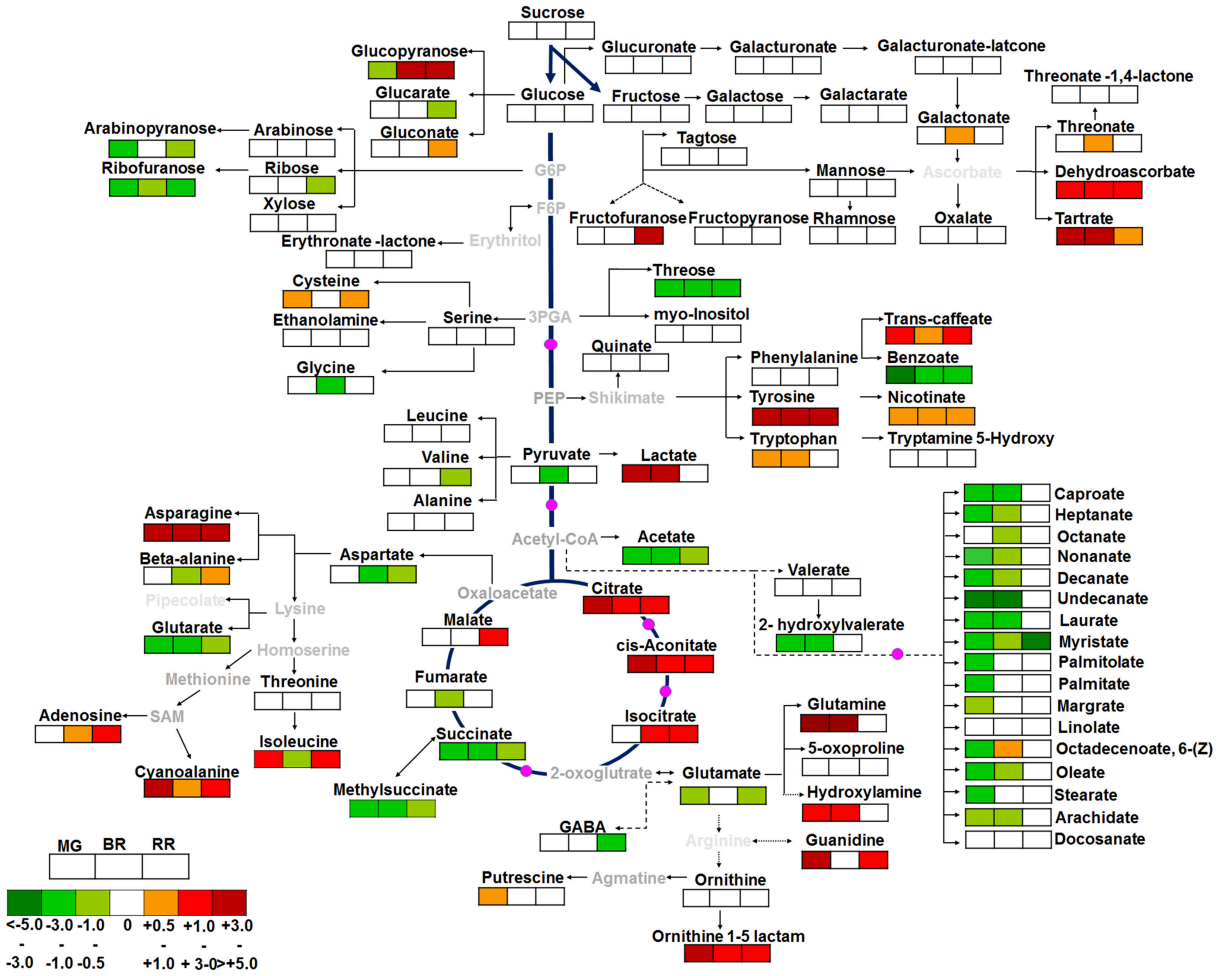
**The metabolic shifts in ***shr*** fruits during ripening in comparison to WT**. The relative changes in the metabolite levels at MG, BR, and RR stage in *shr* fruits were determined by calculating the *shr*/WT ratio at respective ripening phases. Log2-fold changes are represented by varying shades of colors in the boxes with dark red and dark green color representing a maximum increase and decrease respectively at bottom left-hand corner. The white box represents Log2 fold changes in the range of −0.5 to + 0.5. The metabolites in gray letters on pathway were not detected in GC-MS analysis. The pink colored circle on pathways denotes the enzymes reported to be modulated by NO in literature. The values are the mean ±SD (*n* = 3–5 fruits). The metabolites which level were higher or lower to log2FC (−0.5 or +0.5) and statistically significant (Supplementary Table 10) in *shr* in comparison to WT were only showed on pathways.

A total of 96 metabolites were identified in WT and *shr* fruits at three ripening stages, MG, BR, and RR. The levels of several metabolites in *shr* fruits were significantly different from WT at one or more stages (Supplementary Table 10). Principal component analysis (PCA) and the correlation variances explained by the two principal components clearly revealed two clusters of WT and *shr* metabolites (Figure 5). Based on chemical nature the metabolites were grouped as amino acids and amines, sugars, organic acids, fatty acids, and miscellaneous (Supplementary Table 10). Only those metabolites which showed up-regulation or down-regulation >1.5-fold (Log2 *shr*/WT value 0.5) in *shr* fruits than WT were mapped on the metabolic networks (Figure 6, Supplementary Figures 4A–E).

### *shr* mutation preferentially stimulates tyrosine accumulation in fruits

In *shr* fruits, only 14 out of 25 amino acids/amines were differentially regulated at one or more stages. Among these, tyrosine was detected only in *shr* fruits, and its levels progressively declined during ripening. The upregulation of asparagine, tryptophan, alanine 3-cyano, and ornithine 1-5-lactam in *shr* fruits was discernible at all stages with maxima at BR (Figure 6, Supplementary Table 10). The glutamine level in *shr* fruits was significantly higher at MG and BR but was similar to WT at RR. Contrastingly, glutamate was downregulated in *shr* fruits, most significantly at MG and RR (Figure 6, Supplementary Table 10). The hydroxylamine consistently showed higher levels in *shr* fruits. The amino acids derived from the 3-phosphoglycerate and pyruvate showed no or little change in *shr* fruits. Polyamine, putrescine showed significantly high level at MG stage. Considering tyrosine, asparagine, and glutamine showed substantial upregulation (~ 10-fold) and glutamate showed downregulation, these amino acids may have a key role in cellular homeostasis of *shr* fruits.

A total of 27 sugars and their derivatives were identified in *shr* and WT fruits; however only a few were up- or down-regulated in *shr* fruit (Figure 6, Supplementary Table 10). The glucose 6-phosphate derived metabolite ribofuranose was downregulated at all stages in *shr* fruits. Similarly, arabinopyranose and threose (immediate precursor glycerol-3-phosphate) were downregulated in *shr* fruits. While glucopyranose levels were high at BR and RR in *shr* fruits, it was undetectable in WT at same stages.

### *shr* mutation upregulates tricarboxylic acid (TCA) pathway metabolites

In *shr* fruits, out of 6 TCA cycle components, citrate and cis-aconitate were upregulated while succinate and methyl succinate were downregulated at all stages. Isocitrate at BR, RR, and malate at RR were upregulated, and fumarate was downregulated at BR. Lactate increased considerably at MG and BR. Acetyl-CoA derived compound- acetate significantly declined during ripening (Figure 6). Interestingly, dehydroascorbate dimer and tartarate were upregulated at all stages. Caffeate (a chlorogenic acid metabolites) and nicotinate, derived from the shikimate pathway considerably increased at all stages. In addition to TCA cycle components nucleic acid metabolites; guanidine and adenosine were also upregulated at MG-RR and BR-RR stage respectively (Figure 6, Supplementary Table 10).

### *shr* mutation retards fatty acid metabolism during ripening

Interestingly in *shr* fruits, all fatty acids were significantly downregulated at MG and BR except myristate that was downregulated at all stages. Only linolate exhibited no alterations in *shr* compared to WT. During ripening, free fatty acid metabolites in WT progressively declined from a high level at MG, while in *shr* though the level was half of WT, it remained unchanged during ripening.

### *shr* mutation regulates ripening by modulating auxin and abscisic acid level

**Figure 7:**
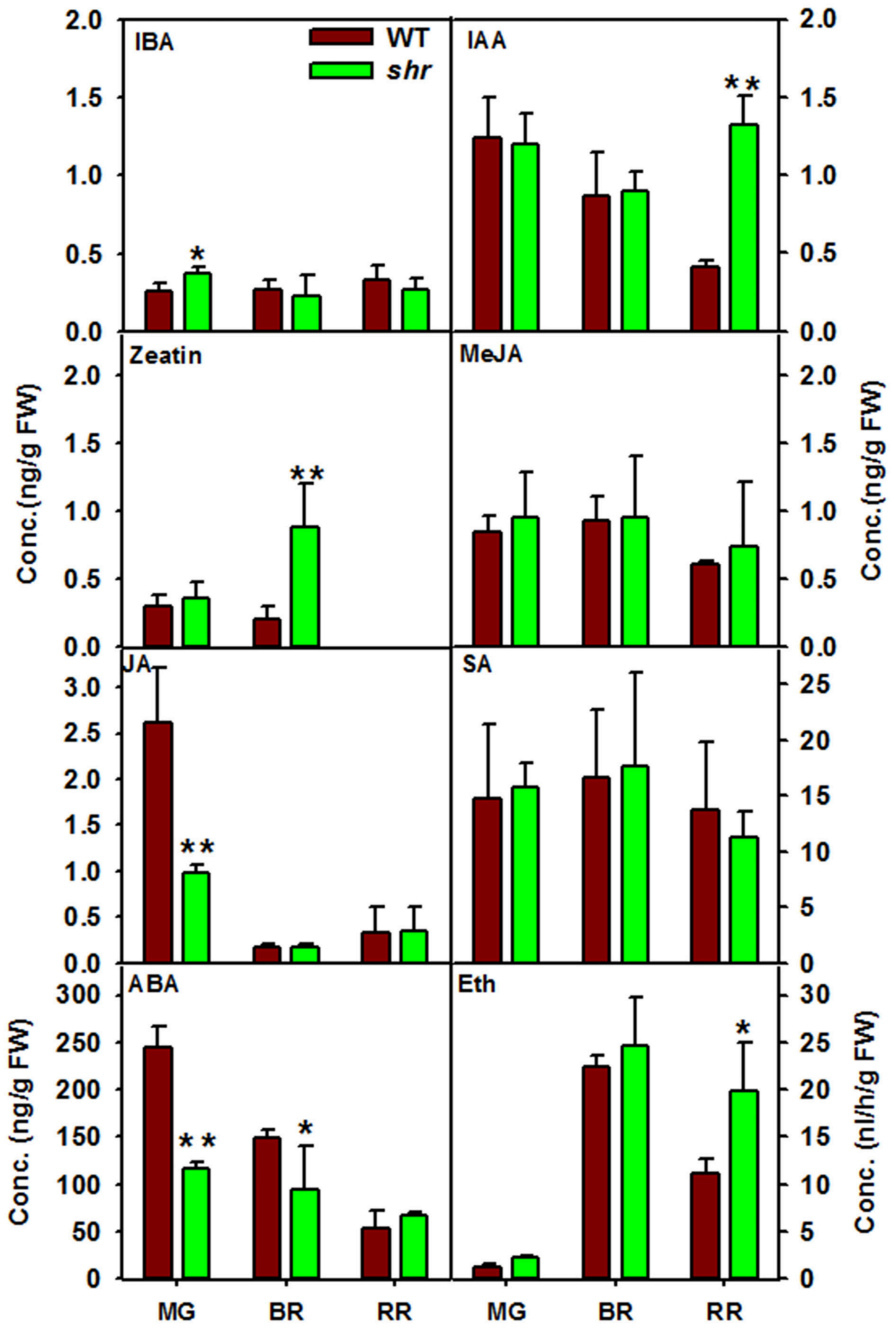
**Phytohormones level in WT and ***shr*** at different stages of fruit ripening**. The levels of all hormones except ethylene were estimated using Liquid Chromatography-Mass Spectrometry (LC-MS). The ethylene was measured by Gas Chromatography (GC). Asterisk indicates statistically significant difference between WT and *shr* (*n* = 3 ±SD; One- Way ANOVA ^*^ < 0.05, ^**^ *P* ≤ 0.001. ABA (Abscisic acid), SA (Salicylic acid), IAA (Indole -3- acetic acid), IBA (Indole-3- butyric acid), JA (Jasmonic acid), MeJA (Methyl Jasmonic acid), Eth (ethylene).

Among the plant hormones, GA and Epi-BR were below the detectable level, and SA, zeatin, MeJA, and JA levels were similar in WT and *shr* fruits. In *shr* fruits, ABA level was low at MG and BR but attained level similar to WT at RR (Figure 7). Conversely, IAA content was high at BR and RR whereas IBA was high at MG in *shr* fruits. The *shr* fruits also emitted higher ethylene at RR (Figure 7). These results indicated that *shr* mutation influenced the temporal changes in ethylene, auxins, and ABA during ripening.

### Metabolites and hormones regulatory network analysis

**Figure 8:**
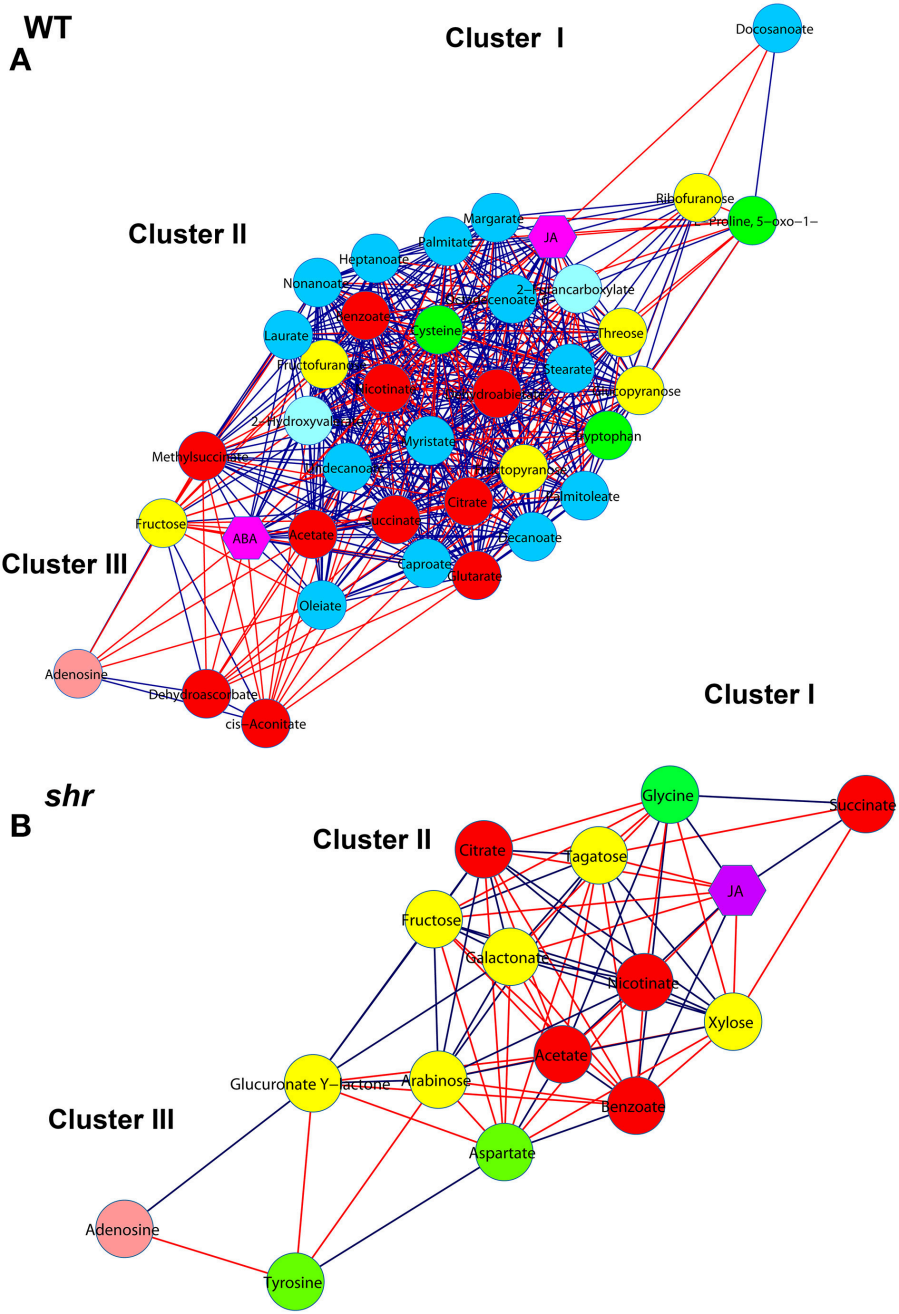
**Correlation network of metabolites and hormones during fruit ripening**. The networks were constructed for WT **(A)** and *shr* **(B)** at MG, BR, and RR stages by including only significantly varying (log2-fold ≤ −0.5, ≥ +0.5) metabolites and hormones. The nodes are represented by different colored circles for metabolites and purple colored hexagons for hormones (ABA and JA). The edges are represented with blue lines for positive correlations and red lines for negative correlations. Statistically significant differences in the level of metabolites across the ripening stages of WT and *shr* were determined throughOne-Way ANOVA (*P* < 0.05).Green nodes, amino acids; yellow nodes, sugars; red nodes, organic acids; blue nodes, fatty acids; light pink nodes, nucleotides.

The regulatory network involved in *shr* fruit ripening was identified by constructing correlation network of significantly different (*P* < 0.05) metabolites and hormones at all stages. The network comprised of 28 metabolites and 2 hormones (ABA and JA) for WT and 16 metabolites and hormone JA for *shr*. In both WT and *shr* network, 3 clusters (I, II, and III) could be distinguished (Figure 8) of which cluster II was most dense with a maximum node connectivity while cluster I and III were sparse with less connectivity.

The PCA of *shr* and WT revealed that the collective complement of metabolites in *shr* fruits was distinctly different from the WT at all stages of fruit ripening. Consistent with this the correlation network of *shr* fruits was distinctly different from WT. First, the network density in *shr* fruits (0.65) was less than the WT (0.71) (Supplementary Table 11). The number of interactions in *shr* was about 1/4th of the WT. Unlike in WT where positive and negative interactions were about 329 and 170 respectively, these were nearly equal in *shr* (+37 and −41). Most importantly there was only a little overlap in the interactions between WT and *shr*. Among 78 interactions that were present in *shr* only 14 were common with WT and positive to the WT. Moreover, WT network showed 31 unique nodes and had only 9 nodes common with *shr*. Similarly, *shr* also showed 8 unique nodes in its correlation network.

These differences between WT and *shr* indicate that the *shr* mutation causes a massive shift in metabolic interaction during the fruit ripening. Most of the interactions that were present in WT were not observed in *shr*. In addition, *shr* showed several unique interactions that were not present in WT. For several metabolites, the interactions were opposite in nature, for example, the interaction of tyrosine with other metabolites (Supplementary Table 11). Interestingly, most of the fatty acids metabolites showed negative interaction with group I (citrate and cis-Aconitate) and positive interaction with group II (acetate, methylsuccinate, and succinate) in WT network, while none of the fatty acids metabolites showed interaction with group I and II metabolites in the *shr* network. These results indicated the metabolites were regulated in a different fashion in *shr* fruits than in the WT.

Examination of WT and *shr* network revealed that TCA pathway metabolites (citrate, cis-aconitate, succinate, methylsuccinate, and acetate) were interconnected and also had maximum connectivity with the other metabolites mostly positioned in cluster II (Figure 8, Supplementary Table 11). On the basis of interactions, two groups were discernible in WT and *shr*. In WT the group I (citrate and cis-acotinate) positively correlated with each other and negatively correlated with group II (succinate, methylsuccinate, and acetate) and vice-versa. Similarly, in *shr* the group I (citrate) negatively correlated with group II (succinate, and acetate) and vice-versa. The phytohormones ABA and JA showed maximum connectivity with cluster II (Figure 8). In WT, ABA, and JA positively correlated with group II and negatively correlated with group I. Similarly in *shr*, JA negatively correlated with the group I and positively with group II.

In both WT and *shr*, the cluster I and III were populated with few metabolites. The tyrosine amino acid that specifically is accumulated at a high level in *shr* fruits was positioned in cluster III and it positively correlated with aspartate (Figure 8, Supplementary Table 11). However, tyrosine negatively correlated with most metabolites in *shr* fruit. In addition, several significantly different out-class metabolites were identified that were present only in *shr* network. Taken together the network analysis indicated that the *shr* mutation distinctly influences the regulation of metabolites during fruit ripening.

## Discussion

### Mapping of *shr* locus and candidate gene prediction

The genetic analysis of *shr* segregation indicated that the *shr* locus is encoded by a single recessive gene located on chromosome nine and it co-segregates with hyperaccumulation of NO.

Using the advantage of the availability of the complete genome sequence of tomato, we overlaid the *shr* locus on to the tomato physical map. The *shr* locus was located within 4.89 Mb (4894020 bp) region of genome scaffold SL2.40ch09:54045071.58939091 (http://solgenomics.net/gb2/gbrowse/ITAG2.3_genomic). Among the known genes regulating NO levels in plants, only one gene was found in the region encompassing *shr* locus. In Arabidopsis, the null alleles of the *HOT5* locus (GSNOR1/HOT5/PAR2) show increase in *in vivo* levels of NO (Lee et al., 2008; Chen et al., 2009). Based on their reported role in regulating NO level in the mammalian system, glutathione S-transferase (Lok et al., 2012) and CUE domain containing protein (Li et al., 2008) were also examined as potential candidate genes. However, these three most obvious candidate genes did not show a mutation in their respective ORFs. Considering that the tomato genome sequence encompassing *shr* locus region has two major unfilled gaps of 35116 bp and 31568 bp size, it could be possible that these gaps may have additional genes and one of them may be encoding for *shr* mutation. Since *shr* mutant was obtained from γ-irradiated population, the possibility remains that rather than a single gene mutation, the chromosomal rearrangement, and/or deletion may have contributed to the phenotype attributed to *shr* locus.

### *shr* mutation retards growth and development

Although the source of *in vivo* NO production (Domingos et al., 2015) remains to be fully deciphered, endogenous NO regulates several facets of higher plant development. The observed diminutive size, sluggish growth and delayed life cycle of the *shr* mutant is consistent with the reports that high endogenous NO level reduces the growth and prolongs the life cycle (Morot-Gaudry-Talarmain et al., 2002). One distinct effect of *shr* locus was on the onset and progression of the reproductive phase. In Arabidopsis, NO overproducer mutant, *nox1* shows delayed flowering (He et al., 2004) whereas NO under-producer mutant *nos1/noa1* shows earlier flowering (Guo et al., 2003). Consistent with this, *shr* mutant displayed delayed development of inflorescence(s) with smaller and fewer flowers than the parental WT.

### Delayed ripening of *shr* fruits may be due to alteration in phytohormone levels

Compared to vegetative development, little is known about the role of endogenous NO in fruit development and quality. So far the information is largely derived by the application of exogenous NO donors to detached fruits with an aim to extend the postharvest shelf life (Manjunatha et al., 2010; Lai et al., 2011). Post-anthesis, the fruit development in *shr* was sluggish with 5–7 days delay in attaining MG stage than the WT. Consistent with the reduction in root and leaf size due to high endogenous NO levels, the MG fruits of *shr* mutant too were half in size than the WT. Even post-MG stage, the transition to different ripening stages was much slower in *shr* fruits than the WT. Attainment of RR stage in *shr* fruit was delayed by ca. 9 days compared to WT. Though hyperaccumulation of NO slowed ripening of fruits, the on vine shelf life of *shr* fruit post-RR stage the was similar to WT. Considering that the carotenoids levels, firmness, and brix of *shr* fruits were similar to WT, it can be assumed that these responses were not affected by NO hyperaccumulation.

Tomato being a climacteric fruit, its ripening is strongly enhanced by the emission of the plant hormone ethylene before the onset of the ripening. The reduction in ethylene biosynthesis by transgenic means also delays tomato ripening (Oeller et al., 1991). Considering that *shr* fruits emitted a higher amount of ethylene than WT, the post MG-delay in ripening is apparently not linked to ethylene biosynthesis. Moreover, our results are not in conformity with the reports that NO downregulates ethylene biosynthesis (Eum et al., 2009; Lai et al., 2011), presumably by S-nitrosylation-mediated inhibition of enzymes regulating ethylene synthesis (Abat and Deswal, 2009). Conversely, our results indicate that higher endogenous NO likely extends the shelf life by delaying the ripening process from MG to RR stage. In several species such as banana, tomato, and strawberries, the application of NO donor SNP (sodium nitroprusside) to detached fruits extended postharvest life (Manjunatha et al., 2010, 2012; Lai et al., 2011). It can be surmised that exogenous NO donors may be extending the fruit shelf life by delaying the overall ripening process.

Apart from their antagonistic interactions in several developmental processes of plants, ethylene and ABA, synergistically promote the ripening process in climacteric fruits (Sun et al., 2012). ABA acts as a principal signal for the onset of ripening, and a decline in ABA levels precedes the climacteric ethylene production in tomato fruit. Considering that the *shr* mutation upregulated ethylene emission at RR stage, it may have affected the endogenous ABA levels. Consistent with this ABA levels in *shr* fruit at MG and BR stages were lower than the WT. The smaller size of *shr* fruits appears to be related to lower ABA levels as ABA deficiency in tomato leads to a reduction in fruit size (Galpaz et al., 2008; Nitsch et al., 2012; Sun et al., 2012). Tomato fruits harvested at the pink stage from ABA deficient plants showed significantly extended shelf life (Sun et al., 2012). Analogously, the slower development of *shr* fruits and prolonged post-MG ripening period is likely related to reduced ABA levels. However, unlike ABA-deficient plants (Galpaz et al., 2008; Sun et al., 2012), carotenoids levels and firmness is not higher in *shr* fruits. Thus, the observed effects of NO on above processes can also arise from a mechanism other than ABA.

In tomato fruits, the endogenous level of free IAA massively declines before the onset of ripening at MG stage followed by a minor rise at RR stage (Böttcher et al., 2010). Contrarily IAA level declined in WT fruits post-MG stage, whereas in *shr* fruits it increased at RR stage. Conversely, *shr* fruits showed higher IBA levels at MG stage than WT. While the role of IBA *per se* is not yet established in fruit ripening, it is well established that auxin-mediated gene expression strongly influences the ripening process, and excess auxin levels cause parthenocarpy in tomato fruits (de Jong et al., 2009). Tomato WT/*35S::IPT* plants showed 1.5-2 fold higher zeatin levels in ripe fruit accompanied with higher fruit weight (Ghanem et al., 2011). Though *shr* fruit had 4-fold higher levels of zeatin than WT, it had no effect on fruit weight. While MeJa level in *shr* was nearly similar to WT, it had nearly 4-fold less JA level at MG stage. Though JA-deficient tomato mutants show a reduction in lycopene level (Liu et al., 2012), *shr* fruit showed no such decline in lycopene. It remains to be established how *shr* mutation affected multiple hormonal responses during ripening. However, the observed changes may result from cross talk between NO and phytohormone(s) as NO is the part of signal transduction chain triggered by several hormones. Such a cross talk has been recently reported in developing tomato fruits where AUXIN RESPONSE FACTOR 2A homodimerizes with ABA STRESS RIPENING (ASR1) protein, thus linking ABA and ethylene-dependent ripening. (Breitel et al., 2016).

### The *shr* mutation likely affects metabolome by modulating TCA cycle

Tricarboxylic acid (TCA) cycle, at the center of cellular metabolism, is interconnected to wider metabolic network contributing to a plethora of pathways such as amino acid biosynthesis (Mackenzie and McIntosh, 1999), regulation of carbon/nitrogen balance (Noguchi and Terashima, 2006), isoprenoid synthesis (Fatland et al., 2005) and cellular redox control (Scheibe et al., 2005) etc. The profiling of proteins from capsicum fruit exposed to NO revealed nitrosylation of a substantial number of enzymes involved in photosynthesis, glycolysis, oxidative/redox metabolism, amino acid biosynthesis, and proteolysis (Chaki et al., 2015). Therefore, it can be presumed that the increased level of NO in *shr* mutant alters the cellular homeostasis by modifying the activity of enzymes involved in metabolic pathways, consequently affecting the fruit size and prolonging the ripening of fruits. This presumption is in consonance with a previous report wherein enhanced levels of central carbon metabolites are associated with reduced fruit size in tomato (Schauer et al., 2006).

Considering that the observed shifts in metabolite levels may arise from multiple factors, we focused only on those metabolites that were significantly different in *shr* from WT at all three stages of fruit development. In *shr* fruits among the intermediates of TCA cycle, the citrate and cis-aconitate levels were high and succinate and its derivative, methylsuccinate were low. In tobacco leaf extracts addition of a NO donor inhibited aconitase activity by forming a metal-nitrosyl complex with the Fe-S cluster of the enzyme (Navarre et al., 2000). The higher levels of TCA cycle intermediates in *shr* fruits appears to be related to NO-mediated inhibition of aconitase activity. The leaves of aconitase deficient mutant of *Lycopersicon pennellii* (*Solanum pennellii*) show a similar increase in citrate levels (Carrari et al., 2003). Likewise, hypoxia induced NO accumulation in Arabidopsis roots concomitantly reduced aconitase activity and increased the citrate and malate levels (Gupta et al., 2012). Similarly, a reduction in aconitase activity in a tomato introgression line increased citrate levels and reduced succinate level in fruits (Morgan et al., 2013). Thus, it can be assumed that enhanced citrate and reduced succinate levels in *shr* fruit may have resulted from inhibition of aconitase. Considering that the tomato non-ripening mutants *rin, Nr*, and *nor* also display reduced level of succinate during ripening (Osorio et al., 2011), the reduced succinate level in *shr* may be linked to prolonged ripening period. However, nitric oxide also affects the activity of other TCA cycle constituents; succinate dehydrogenase (Simonin and Galina, 2013) and cytochrome C oxidase (Millar and Day, 1996). The observed shift in TCA cycle intermediates and ensuing metabolome may thus represent a cumulative effect of NO on a plethora of enzymes and proteins. From the foregoing, it is apparent that the reduction in fruit size and prolonged ripening of *shr* fruits may have a relationship with alteration in central carbon metabolism.

### The cellular aminome is altered in *shr* fruits

The *shr* mutation had a broad spectrum effect on the cellular aminome eliciting significant changes in levels of several amino acids during fruit ripening. The NO-mediated aconitase inhibition reportedly activates the alternate oxidase pathway and shifts the metabolism toward upregulation of amino acids (Gupta et al., 2012). Among the upregulated amino acids, the high level of hydroxylamine in *shr* fruits may have a relationship to NO biosynthesis as tobacco cell suspensions reportedly convert hydroxylamine to NO (Rümer et al., 2009). Little is known about the role of 3-cyanoalanine in fruit ripening except that it is a byproduct in detoxification of HCN produced during ethylene emission from fruits. Though ornithine,1-5 lactam levels were higher in *shr* fruits, it had no significant effect on polyamines levels which are implicated for longer shelf life of fruits (Mehta et al., 2002), except putrescine at MG stage.

The strong upregulation of tyrosine in *shr* fruit is intriguing. The increased level of tyrosine may signify a block in its downstream metabolism or strong upregulation of its biosynthesis. Considering that the activity of arogenate dehydrogenase that converts arogenate to tyrosine is strongly inhibited by tyrosine (Rippert and Matringe, 2002), the upregulation of biosynthesis is unlikely. Alternately tyrosine can be synthesized from prephenate by the action of prephenate dehydrogenase which lacks feedback regulation by tyrosine via 4-hydroxyphenylpyruvate (Schenck et al., 2015). However, this pathway is reported only in legumes. Nonetheless, upregulation of tyrosine level represents a very specific modulation of a metabolite level by *shr* mutation.

Considering that asparagine is derived from glutamine and aspartate, the high level of glutamine may have correspondingly increased the asparagine levels, indicating a co-ordinated upregulation of these two amino acids. This is also corroborated by the reduced level of aspartate in *shr* fruits. In tomato, arbuscular mycorhizzal association specifically upregulates asparagine and glutamine levels in fruits, presumably by promoting their transport from root to the fruits (Salvioli et al., 2012). Considering that *shr* mutation strongly influences the root phenotype, it may have also influenced the mobilization of these amino acids to the fruit. The reduced level of the glutamate may have a relationship with the prolonged period of ripening of *shr* fruit. A comparison of glutamate levels in *rin* and *nor* non-ripening mutants of tomato with a normal cultivar revealed a significant negative correlation between fruit glutamate levels and shelf life, with lower glutamate levels being associated with a longer shelf life (Pratta et al., 2004). Thus, the lower level of glutamate in *shr* fruit is consistent with its slower ripening.

The progression of fruit ripening in tomato is associated with a steady decline in the fatty acid levels. However, in *shr* fruits, the levels of most fatty acids were much lower even at MG stage and for some even at BR stage. By RR stage, due to a continual decline in fatty acids levels in WT, their levels became nearly equal to *shr* fruits. In leaves of Arabidopsis *ssi2* mutant, the reduction in oleic acid (18:1) level has been shown to induce NO production in chloroplast (Mandal et al., 2012). Considering that level of oleate in *shr* fruits is lower it may have a linkage with the *shr* mutation.

### *shr* mutation shifts cellular homeostasis

The sizable shift in metabolomic interactions, loss of nodes present in WT and appearance of new nodes in *shr* likely reflects a broad spectrum action of *shr* mutation. A large number of proteins regulating a range of metabolic and developmental processes are known to be targets of NO (Hu et al., 2015). Assuming that the observed shift is related to hyperaccumulation of NO in *shr* mutant, it is plausible that it may have affected the activity of several key proteins regulating cellular metabolism. This presumption is consistent with the report that exogenous application of NO to pepper fruits delayed fruit ripening which may be related to protein nitration of key enzymes (Chaki et al., 2015). One of the distinct responses related to protein nitrosylation pertains to ABA signaling. Plants deficient in NO are hypersensitive to ABA and tyrosine nitration of ABA receptor by NO inhibits ABA signaling (Castillo et al., 2015). The absence of ABA in the correlation network of *shr* fruits may reflect such a negative effect of the *shr* mutation on ABA-triggered signal transduction. The effect of NO was not restricted to ABA alone. Though JA mapped on the *shr* network, it interacted with a different metabolite sets than in WT.

Currently, little is known how developmental mutants regulate the metabolic shifts. Similar to *shr* mutant, tomato *sun* mutant also showed massive shifts in metabolite interactions, with the loss of several interactions and appearance of unique interactions compared to WT (Clevenger et al., 2015). While *shr* had 64 unique interaction pairs and lost 485 interaction pairs present in the WT, *sun* fruits had 151 unique interaction pairs and lost 273 interaction pairs. Consistent with SUN being a protein with calmodulin recruitment domains, the mutation in it affects the calcium related processes; the major metabolic shifts in *sun* mutant were related to calcium signaling (Clevenger et al., 2015). Likewise, it can be assumed that analogous to *sun* mutant, the metabolic shifts in *shr* mutant may be related to its modulation of NO level. The shift in *shr* correlation networks probably stems from a requirement to sustain the metabolomic homeostasis affected by the *shr* mutation. The altered interactions between different metabolites likely arise from the need to maintain the cellular homeostasis to continue the normal process of ripening (Fares, 2015; Ho and Zhang, 2016), though the overall duration of ripening in *shr* is prolonged. While it can be presumed that the observed loss and gain of metabolite interactions in *shr* represents the process of metabolic compensation, the mechanisms underlying this process are yet to be deciphered.

In summary, the characterization of *shr* mutant indicated that hyperaccumulation of NO slows the on-vine process of fruit ripening in tomato, possibly by altering the overall cellular homeostasis. Our results have an implication for increasing the shelf life of tomato, as selective manipulation of NO levels during ripening can keep the fruits fresh for a longer duration.

## Authors contributions

The crosses for mapping were made by KS. The mapping analysis of F_2_ plants was done by RB and PB. The fruit and seedling phenotyping and metabolic characterization were done by RB and SG. The candidate gene prediction was done by RS and YS. Overall conceptualization of work was done by RS. RB, SG, YS, and RS were involved in writing of manuscript. S and SG made the correlation networks. All authors read and approved the manuscript.

### Conflict of interest statement

The authors declare that the research was conducted in the absence of any commercial or financial relationships that could be construed as a potential conflict of interest.

## Supplementary material

Supplementary Figure S1 **The *shr* mutant plants showed sluggish growth**. 45-day-old greenhouse grown *shr*, WT, *S. pimpinellifolium* (*SP*), and F_1_ plants were compared **(A)** Morphology of plants. **(B)** Internode (5–6th) length (*upper panel*) and height (*lower panel*). **(C)** Variation in leaf size and morphology. The leaves were harvested from the 7th node of respective plants. **(D)** Chlorophylls and carotenoids levels. Asterisk indicates statistically significant difference between WT and *SP, shr* and F_1._ The values are the mean ±SD (*n* = 5). Asterisk indicates statistically significant difference between WT and *SP, shr*, and F_1_ (One Way ANOVA ^*^ <0.05, ^**^*P* < 0.001).

Supplementary Figure S2 **Bulk segregant analysis (BSA) and genotyping of F_2_ mapping lines**. PCR amplification profile of bulk segregants with the TGS0213 **(A)** and C2_At3g63190 markers **(B)**. P1, *short root* parent; P2, *S. pimpinellifolium*; B1, short root DNA bulk; B2, long root DNA bulk. **(C)** PCR-based genotyping of TGS0123 marker using *shr* x *S. pimpinellifolium* F_2_mapping population. The lanes 1–22 are F_2_ mapping population individuals. Lanes 1–6 are *shr* individuals, and lanes 7–22 are other than short root. The lanes 7–9, 11, 13–15, and 17–20 are heterozygotes. The lanes 10, 12, 16, and 21–22 are long root individuals. The PCR products were electrophoresed on 3.5% (w/v) agarose gel. M- 100-bp DNA ladder.

Supplementary Figure S3 **Ripening induced changes in WT and *shr* mutant at different stages of ripening. (A)** Brix content. **(B)** Fruit firmness. **(C)** Fruit pH at RR stage. Asterisk indicates statistically significant difference between WT and *shr* mutant (mean ±SD; *n* = 5, Student's *t*-test ^*^ *P* ≤ 0.05).

Supplementary Figure S4 **Relative levels of different metabolites in *shr* and WT**. The relative level of metabolites was obtained by dividing the peak area of ribitol, the internal standard. Data are the mean value of n ≥ 3 ± S.D. (One Way ANNOVA ^*^ *P* < 0.05, ^**^ *P* ≤ 0.001). Only most significant metabolites are presented here, the list of total metabolites is given in Supplementary Table 10. **(A)**, organic acids; **(B)**, Amino acids; **(C)**, Sugars; **(D)**, Fatty acids; **(E)**, miscellaneous compounds. MG, mature green; BR,breaker; RR, red ripe.

Supplementary Table S1 **The details of SSR and Indel markers used for mapping of *shr* locus**. (http://solgenomics.net/ and http://www.tomatomap.net/, Last accessed in 2013).

Supplementary Table S2 **List of additional Simple Sequence Repeats (SSR) markers selected from Kazusa DNA research institute (http://marker.kazusa.or.jp/tomato)**.

Supplementary Table S3 **List of additional Simple Sequence Repeats (SSR) markers selected from Veg Marks, a DNA marker database for vegetables (http://vegmarks.nivot.affrc.go.jp, Last accessed in 2013)**.

Supplementary Table S4 **List of Cleaved Amplified Polymorphic Sequences (CAPS) markers used to map *shr* locus on the chromosome (http://solgenomics.net)**.

Supplementary Table S5 **The genetic segregation of short root phenotype**. The segregation was analyzed in the progeny of *shr* x *S. pimpinellifolium* and WT x *shr*. The seedlings were grown under white light and segregation of short root, and long root phenotype in F_1_ and F_2_ generation was analyzed 7–9 days after germination.

Supplementary Table S6 **The segregation of lateral root phenotype in the progeny of *shr* x *S. pimpinellifolium***. The seedlings were grown under white light and segregation of lateral root, and no lateral root phenotype in F_1_ and F_2_ generation was analyzed 7–9 days after germination. The identical segregation ratio for lateral root was obtained for another tomato mutant in Ailsa Craig background that was crossed with *S. pimpinellifolium* (data not shown) indicating that lateral root gene was contributed by *S. lycopersicon* and was unrelated to *shr* locus.

Supplementary Table S7 **Genotype frequency for molecular markers on chromosome 9 in the mapping population derived from *shr* x *S. pimpinellifolium***.

Supplementary Table S8 **List of genes between SSR marker TGS0213 and CAPS marker C2_At3g6310 (http://solgenomics.net/gb2/gbrowse/ITAG2.3_genomic/)**. Expression of genes was retrieved from Tomato Genome Consortium (2012).

Supplementary Table S9 **Carotenoids content in fruits of WT and *shr* mutant at mature green (MG), breaker (BR) and red ripe stage (RR) stages of ripening**.

Supplementary Table S10 **List of metabolites identified in MG, BR, and RR stage of WT and *shr* fruits by GC-MS**.

Supplementary Table S11 **List of metabolites interactions in WT and *shr* correlation networks**. The metabolites present in WT and *shr* networks, shared metabolites, their interaction with hormone and fatty acids is also presented.
