# Supplementary figures and images for: Nitric Oxide Overproduction in Tomato shr Mutant Shifts Metabolic Profiles and Suppresses Fruit Growth and Ripening
Source: Front Plant Sci. 2016 Nov 28;7:1714. doi: 10.3389/fpls.2016.01714 (PMC5124567; doi:10.3389/fpls.2016.01714)

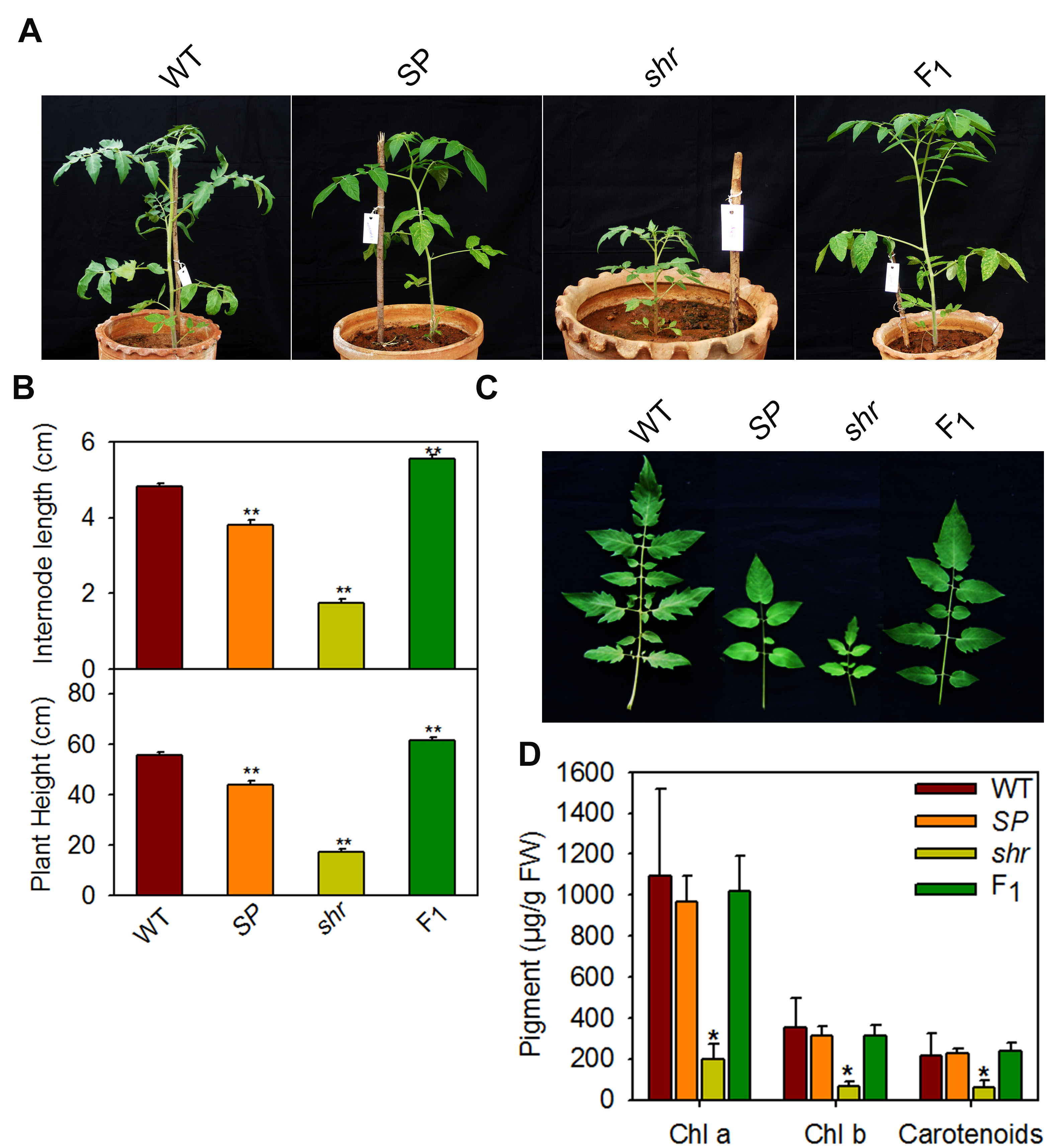

Supplement: Supplementary Figure S1 — The shr mutant plants showed sluggish growth. 45-day-old greenhouse grown shr, WT, S. pimpinellifolium (SP), and F1 plants were compared (A) Morphology of plants. (B) Internode (5–6th) length (upper panel) and height (lower panel). (C) Variation in leaf size and morphology. The leaves were harvested from the 7th node of respective plants. (D) Chlorophylls and carotenoids levels. Asterisk indicates statistically significant difference between WT and SP, shr and F1. The values are the mean ±SD (n = 5). Asterisk indicates statistically significant difference between WT and SP, shr, and F1 (One Way ANOVA * <0.05, **P < 0.001). [file Image1.JPEG]

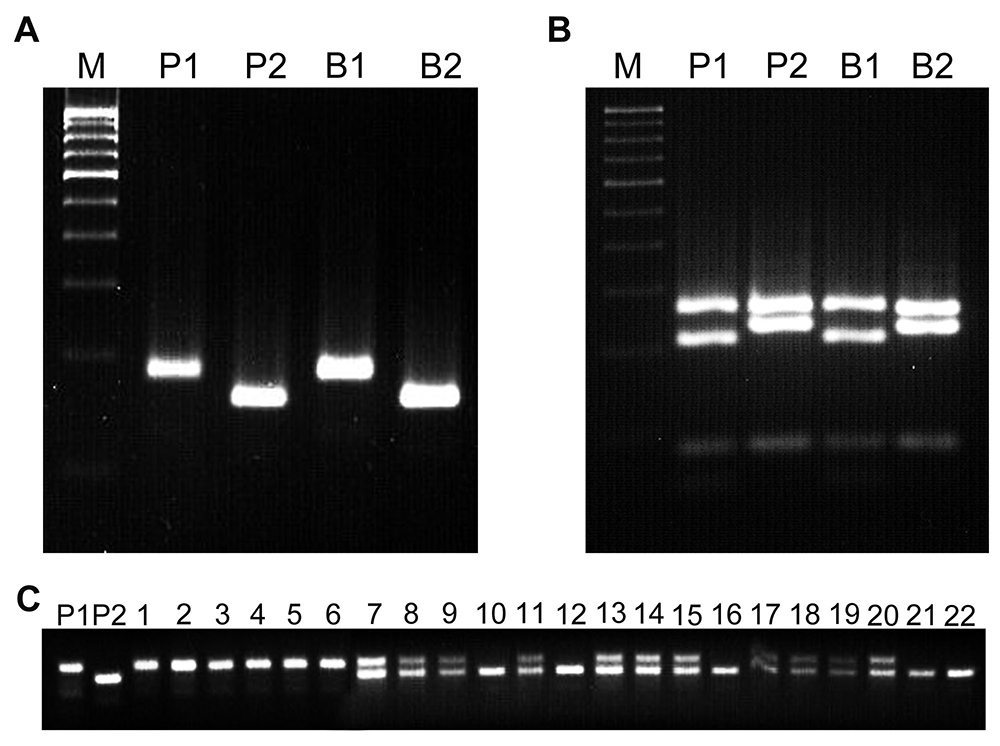

Supplement: Supplementary Figure S2 — Bulk segregant analysis (BSA) and genotyping of F2 mapping lines. PCR amplification profile of bulk segregants with the TGS0213 (A) and C2_At3g63190 markers (B). P1, short root parent; P2, S. pimpinellifolium; B1, short root DNA bulk; B2, long root DNA bulk. (C) PCR-based genotyping of TGS0123 marker using shr x S. pimpinellifolium F2mapping population. The lanes 1–22 are F2 mapping population individuals. Lanes 1–6 are shr individuals, and lanes 7–22 are other than short root. The lanes 7–9, 11, 13–15, and 17–20 are heterozygotes. The lanes 10, 12, 16, and 21–22 are long root individuals. The PCR products were electrophoresed on 3.5% (w/v) agarose gel. M- 100-bp DNA ladder. [file Image2.JPEG]

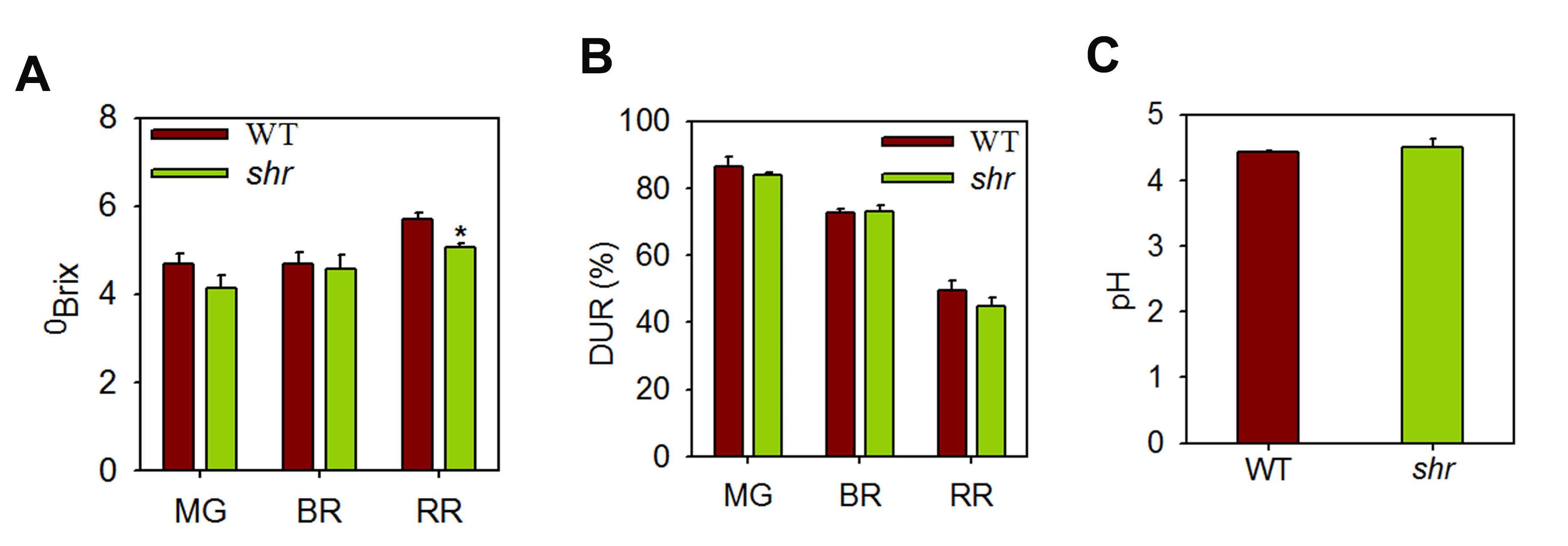

Supplement: Supplementary Figure S3 — Ripening induced changes in WT and shr mutant at different stages of ripening. (A) Brix content. (B) Fruit firmness. (C) Fruit pH at RR stage. Asterisk indicates statistically significant difference between WT and shr mutant (mean ±SD; n = 5, Student's t-test * P ≤ 0.05). [file Image3.JPEG]

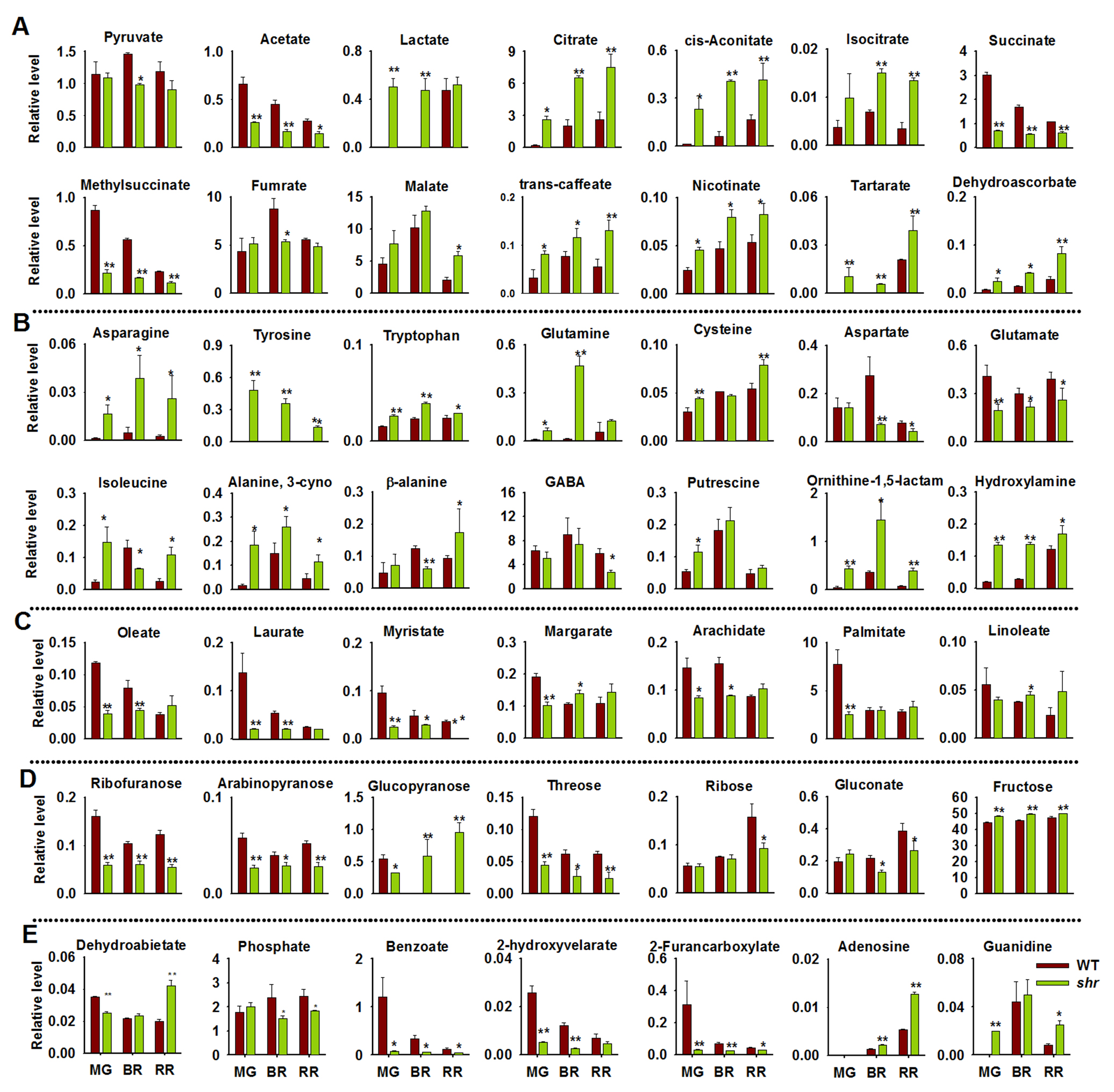

Supplement: Supplementary Figure S4 — Relative levels of different metabolites in shr and WT. The relative level of metabolites was obtained by dividing the peak area of ribitol, the internal standard. Data are the mean value of n ≥ 3 ± S.D. (One Way ANNOVA * P < 0.05, ** P ≤ 0.001). Only most significant metabolites are presented here, the list of total metabolites is given in Supplementary Table 10. (A), organic acids; (B), Amino acids; (C), Sugars; (D), Fatty acids; (E), miscellaneous compounds. MG, mature green; BR,breaker; RR, red ripe. [file Image4.jpg]
