# Supplementary material for: Nitric Oxide Overproduction in Tomato shr Mutant Shifts Metabolic Profiles and Suppresses Fruit Growth and Ripening
Source: Front Plant Sci. 2016 Nov 28;7:1714. doi: 10.3389/fpls.2016.01714 (PMC5124567; doi:10.3389/fpls.2016.01714)
Supplement: Supplementary Table S1 — The details of SSR and Indel markers used for mapping of shr locus. (http://solgenomics.net/ and http://www.tomatomap.net/, Last accessed in 2013). [file Table1.DOCX]

**Supplementary Material**

**Nitric oxide overproduction in tomato shr mutant alters cellular homeostasis and suppresses fruit growth and ripening**

*Reddaiah Bodanapu, Suresh Kumar Gupta, Pinjari Osman Basha, Kannabiran Sakthivel, Sadhna, Yellamaraju Sreelakshmi and Rameshwar Sharma*

**Corresponding author:** rameshwar.sharma@gmail.com

**Supplementary Table S1.** The details of SSR and Indel markers used for mapping of *shr* locus. (<http://solgenomics.net/> and <http://www.tomatomap.net/>).

| **S. No** | **Marker name** | **Chromosome** | **Position**  **(cM)** | **Repeat type and length** | **Primer sequence (5'→3')**  **(F: Forward primer, R: Reverse primer)** | **Product size (bp)** |
| --- | --- | --- | --- | --- | --- | --- |
| 1 | SSR478 | 1 | 0.00 | (GGTG)_2_(CT)_16_ | F: GCAGCATATATCACCTTGGCT  R: CGTGCTCTCCAATAGTTCACC | 272 |
| 2 | SSR92 | 1 | 0.00 | (CT)_11_ | F:AAGAAGAAGGATCGATCGAAGA  R:TCATGACCACGATACTACATGTTTC | 172 |
| 3 | TMS45 | 1 | 12.60 | NA | F: CCGTCCAGAAGACGATGTAA  R: CAAAGTCTTGCCAACAATCC | NA |
| 4 | SSR192 | 1 | 31.50 | (ATC)_5_ | F: ACAACATGGGAAGCACTTGA  R: ATTAAATTGGGCCATGGTGA | 168 |
| 5 | SSR61 | 1 | 33.00 | (GA)_24_ | F: ATGCCTTATGGAAACAACGC  R: CGGGTGTACGAATGTCTTTG | 237 |
| 6 | SSR51 | 1 | 39.50 | (ACAA)_6_ | F: CTACCCTGGTCTTGGTGGAA  R: AAAGGATGCTCTAGCTTCTCCA | 148 |
| 7 | SSR270 | 1 | 46.00 | (GAA)_5_(GGAGAA)_7_ | F: AGCTCAAGGCTTCTGTTGGA  R: AACCACCTCAGGCACTTCAT | 231 |
| 8 | SSR316 | 1 | 46.70 | (AG)_6_(TTGCAG)_2_ | F: CCACCGCAACAAACCTTATT  R: GGGTGGTGAGAAGGATCTGA | 215 |
| 9 | SSR75 | 1 | 53.50 | (AAT)_9_ | F: CCATCTATTATCTTCTCTCCAACAC  R: GGTCCCAACTCGGTACACAC | 155 |
| 10 | SL10975i* | 1 | 68.70 | TAAAAGGATG | F: GTGAACCCGGAACTCTGAAC  R: TCATTGCCACACAGAAGCAG | NA |
| 11 | SSR134 | 1 | 75.00 | (TA)_20_ | F: CCCTCTTGCCTAAACATCCA  R: CGTTGCGAATTCAGATTAGTTG | 171 |
| 12 | SSR29 | 1 | 91.00 | (AAG)_7_ | F: AGTTGCTACACAGCAGCCAA  R: AGCGATGAGACAAGAGGCAG | 156 |
| 13 | SSR222 | 1 | 97.50 | (TCT)_7_ | F: TCTCATCTGGTGCTGCTGTT  R: TTCTTGGAGGACCCAGAAAC | 183 |
| 14 | SSR150 | 1 | 111.50 | (CTT)_7_ | F: ATGCCTCGCTACCTCCTCTT  R: AATCGTTCGTTCACAAACCC | 215 |
| 15 | SSR308 | 1 | 121.00 | (TA)_12_ | F: TTTCCCTGTTTCAGCCTTTG  R: GGCACGAGAATTTAGCCACT | 293 |
| 16 | SSR346 | 1 | 125.00 | (CT)_2_(CT)_8_ | F: AAACGCCATATAACACGTTGC  R: TGCTTTCCATAGGATTTCCG | 282 |
| 17 | SSR9 | 1 | 134.00 | (ATA)_10_ | F: CCCTTTGCAAGTTCTTCTTCA  R: TTCATGAGCCAACATAGGAGG | 168 |
| 18 | SSR156 | 1 | 146.00 | (TCT)_7_ | F: CACGCCTATGCACCTTTCTT  R: CTTCAAGGCTAAACCTCCGA | 167 |
| 19 | SSR288 | 1 | 158.00 | (TGA)_7_ | F: TCGTGGGAATTTGTTAACCC  R: TCTTCATCGTCCTCCTCCTG | 275 |
| 20 | SSR65 | 1 | 159.00 | (AG)_5_(TG)_7_ | F: CACGCCTATGCACCTTTCTT  R: CTTCAAGGCTAAACCTCCGA | 230 |
| 21 | SSR448 | 2 | 0.00 | (CT)_14_ | F: GAATGACCATCAGTACGGC  R: CTGTGCTCCTCTATGCCCTC | 163 |
| 22 | TOM11 | 2 | 3.00 | NA | F: ATTGTAATGGTGATGCTCTTCC  R:CAGTTACTACCAAAAATAGTCAAAAC | NA |
| 23 | SSR586 | 2 | 6.00 | (AAC)_6_ | F: TCCATCTAAGGTCTTTGCCG  R: ACAAAGGAAGTGGGAGAGCA | 125 |
| 24 | SSR125 | 2 | 11.00 | (CTAG)_2_(GT)_7_  (CTTT)_4_ | F:CCTAAAGAAGATAGGAAGAAATGCC  R: TCTCTCCTACTGAAACAACCAA | 192 |
| 25 | SSR40 | 2 | 22.00 | (AC)_7_(GC)_7_ | F: TGCAGGTATGTCTCACACCA  R: TTGCAAGAACACCTCCCTTT | 146 |
| 26 | SSR66 | 2 | 25.00 | (ATA)_8_ | F: TGCAACAACTGGATAGGTCG  R: TGGATGAAACGGATGTTGAA | 185 |
| 27 | SSR96 | 2 | 36.50 | (AT)_12_ | F: GGGTTATCAATGATGCAATGG  R: CCTTTATGTCAGCCGGTGTT | 222 |
| 28 | SSR349 | 2 | 47.50 | (ATAAAA)_2_(TA)_11_ | F: GAGTGATCATCCATCCTCTCA  R: GGAAGAGACTTTGGACTAAGGGA | 241 |
| 29 | SSR103 | 2 | 51.00 | (CT)_13_(CCCT)_3_ | F: CCAACTGGATGACCTTTGTG  R: GTCCAGTGTTTCCAAAGGGA | 160 |
| 30 | SSR5 | 2 | 53.00 | (CAA)_6_ | F: TGGCCGGCTTCTAGAAATAA  R: TGAAATCACCCGTGACCTTT | 196 |
| 31 | SSR50 | 2 | 70.50 | (TC)_6_(CCTTC)_2_ | F: CCGTGACCCTCTTTACAAGC  R: TTGCTTTCTTCTTCGCCATT | 241 |
| 32 | SSR26 | 2 | 77.50 | (CGG)_7_ | F: CGCCTATCGATACCACCACT  R: ATTGATCCGTTTGGTTCTGC | 178 |
| 33 | SSR598 | 2 | 78.00 | (TTC)_7_ | F: TGGAAAGAAGCAGTAGCATTG  R: CAACGAACATCCTCCGTTCT | 186 |
| 34 | SSR287 | 2 | 107.00 | (TA)_12_ | F: GCATCCCAAACAATCCAATC  R: TCCACTTTCAAGATCAGAGCAA | 168 |
| 35 | fw 2.2 | 2 | 117.00 | NA | F: GCGAGAGCGAGTTGAGTGTATATC  R: CAGAAGAGAGAAGCTGCAAAGCAG | NA |
| 36 | SSR86 | 3 | 54.00 | (AG)_10_ | F: AGGGCAACAAATCCCTCTTT  R: GGAGACGAGGCTGCTTACAC | 210 |
| 37 | SSR111 | 3 | 73.90 | (TG)_6_(TCTG)_6_ | F: TTCTTCCCTTCCATCAGTTCT  R: TTTGCTGCTATACTGCTGACA | 188 |
| 38 | SSR330 | 3 | 75.50 | (TA)_13_ | F: GCCCAACATTTGCTCAAGTC  R: CCGGTAGGTAGAGAATGAAGACA | 279 |
| 39 | SL10042i***** | 3 | 96.60 | TTATTAGGTAACCATTAATAAAGC | F: ATTTGCCGACACGTCATTTC  R: CACTCATCATGACAAAGACAACC | NA |
| 40 | SSR22 | 3 | 99.00 | (TA)_11_ | F: GATCGGCAGTAGGTGCTCTC  R: CAAGAAACACCCATATCCGC | 217 |
| 41 | T0581 | 3 | 103.00 | NA | F: CGACTGATGTTCGTAAGGCCC  R: AGCGTAGAGCTTGCGTTCACA | NA |
| 42 | SL10772i* | 3 | 104.50 | TTT | F: TGCAAAAACATATCGCAATTC  R: GAGGGTCATTTCAGTAGACAGC | NA |
| 43 | SSR320 | 3 | 158.00 | (AT)_12_ | F: ATGAGGCAATCTTCACCTGG  R: TTCAGCTGATAGTTCCTGCG | 171 |
| 44 | SSR14 | 3 | 162.50 | (ATA)_9_ | F: TCTGCATCTGGTGAAGCAAG  R CTGGATTGCCTGGTTGATTT: | 166 |
| 45 | SSR11 | 3 | 164.00 | (CAG)_6_ | F: CCTTCAATTGACCTCCCTCA  R: GCATCTGGAAATTAGAGGCG | 165 |
| 46 | SSR290 | 3 | 171.00 | (AT)_12_ | F: AATCAAGCCAACGCCTCTAA  R: TCATCCTTCGCATACTTCCC | 171 |
| 47 | SSR72 | 4 | 0.00 | (CAG)_6_ | F: GGTTCCCTTCTCTCTTTGTCC  R: GCGTGTTCTTCGATTTGACA | 165 |
| 48 | SSR43 | 4 | 14.00 | (TAC)_7_ | F: CTCCAAATTGGGCAATAACA  R: TTAGGAAGTTGCATTAGGCCA | 237 |
| 49 | SSR593 | 4 | 15.00 | (TAC)_7_ | F: TGGCATGAACAACAACCAAT  R: AGGAAGTTGCATTAGGCCAT | 295 |
| 50 | U21085 | 4 | 26.10 | NA | F: CATTTTATCATTTATTTGTGTCTTG  R: ACAAAAAAAGGTGACGATACA | NA |
| 51 | SSR60 | 4 | 37.00 | (GAA)_8_ | F: CCTTCAACTTCACCACCACC  R TGTTGGTTGGAGAAACTCCC: | 251 |
| 52 | SSR603 | 4 | 45.29 | (GAA )8 | F:GAAGGGACAATTCACAGAGTTTG  R:CCTTCAACTTCACCACCACC | 251 |
| 53 | SSR450 | 4 | 54.50 | (AAT)_7_ | F: AATGAAGAACCATTCCGCAC  R: ACATGAGCCCAATGAACCTC | 265 |
| 54 | SSR638 | 4 | 58.00 | NA | F: TGTTGGTTGGAGAAACTCCC  R: AGGCATTTAAACCAATAGGTAGC | NA |
| 55 | SSR555 | 4 | 61.50 | (CTT)_9_ | F: TTGATATTAACCATGGCAGCAG  R: TTGATGGGATTGCACAGAAA | 217 |
| 56 | SSR214 | 4 | 95.00 | (CTT)_7_ | F: AAATTCCCAACACTTGCCAC  R: CCCACCACTATCCAAACCC | 221 |
| 57 | TOM184 | 4 | 96.00 | NA | F: CAACCCCTCTCCTATTCT  R: CTGCTTTGTCGAGTTTGAA | NA |
| 58 | SSR146 | 4 | 102.00 | (ATC)_7_(CAT)_5_ | F: TATGGCCATGGCTGAACC  R: CGAACGCCACCACTATACCT | 243 |
| 59 | SSR188 | 4 | 135.30 | (AT)_11_ | F: TGCAGTGAGTCTCGATTTGC  R: GGTCTCATTGCAGATAGGGC | 135 |
| 60 | SSRB60800 | 4 | 137.00 | NA | F: AGATCAAGATTAAGACCACCGGAGC  R: TCAGGTGCGAGTGTTACAATCTCTG | NA |
| 61 | SSR62 | 5 | 10.50 | (TAA)_7_ | F: TGCAAATGAATGTCCAGGAT  R: TCAGCAGAGTTATGCCATGC | 234 |
| 62 | SSR325 | 5 | 18.50 | (TAA)_7_ | F: CCATTGACAGCCCATTATCC  R: TGATGTGAAAGAGTTGATGAGG | 132 |
| 63 | SSR602 | 5 | 27.00 | (AAG)_6_ | F: GGGTCACATACACTCATACTAAGGA  R: GGCAATCATAGCCACTTGGT | 299 |
| 64 | SSR13 | 5 | 28.00 | (AAG)_6_ | F: GGGTCACATACACTCATACTAAGGA  R: CAAATCGCGACATGTGTAAGA | 104 |
| 65 | SSR115 | 5 | 35.00 | (AT)_16_ | F: CACCCTTTATTCAGATTCCTCT  R: ATTGAGGGTATGCAACAGCC | 211 |
| 66 | TOM152 | 5 | 56.00 | NA | F: ATTCAAGGAACTTTTAGCTCC  R: TGCATTAAGGTTCATAAATGA | NA |
| 67 | SSR49 | 5 | 106.00 | (TC)_6_(AC)_4_ | F: TCTCAAAGTCGTTCCTTCTTGA  R: GGAAGAGAAACGCGGACATA | 161 |
| 68 | SSR590 | 5 | 107.50 | (TC)_6_(AC)_4_ | F: TCTCAAAGTCGTTCCTTCTTGA  R: GGAAGAGAAACGCGGACATA | 161 |
| 69 | SSR162 | 5 | 119.00 | (TA)_14_ | F: GCTCTCTACAAGTGGAACTTTCTC  R: CAACAGCCAGGAACAAGGAT | 224 |
| 70 | SSR47 | 6 | 6.500 | (AT)_14_ | F: TCCTCAAGAAATGAAGCTCTGA  R: CCTTGGAGATAACAACCACAA | 191 |
| 71 | SSR128 | 6 | 35.00 | (CAG)_6_(CAA)_3_  (CAG)_7_ | F: GGTCCAGTTCAATCAACCGA  R: TGAAGTCGTCTCATGGTTCG | 123 |
| 72 | SSR326 | 6 | 39.00 | (AG)_10_ | F: TTAGGCAGCTTACGACTGGA  R: GAGCCAGAGGTCCTTCAGTG | 300 |
| 73 | SSR578 | 6 | 44.00 | (AAC)_6_(ATC)_5_ | F: ATTCCCAGCACAACCAGACT  R: GTTGGTGGATGAAATTTGTG | 296 |
| 74 | SSR350 | 6 | 55.00 | (AT)_13_ | F: GGAATAACCTCTAACTGCGGG  R: CGATGCCTTCATTTGGACTT | 267 |
| 75 | SSR122 | 6 | 101.00 | (AG)_3_(AAG)_7_ | F: ACGGGATTGTACCCAATCAA  R: AGGGTTTGAAGAGGAGGAGG | 172 |
| 76 | SSR241 | 7 | 0.00 | (AAT)_13_ | F: TCAACAGCATAGTGGAGGAGG  R: TCCTCGGTAATTGATCCACC | 200 |
| 77 | SSR285 | 7 | 2.00 | (AAT)_13_ | F: AGTGGCTCTCACCTACTGCG  R: CAATTCTCAGGCATGAAACG | 276 |
| 78 | SSR286 | 7 | 12.00 | (CAG)_8_ | F: AGCTATGGAGTTTCAGGACCA  R: ATTCAGGTAGCATGGAACGC | 107 |
| 79 | SSR276 | 7 | 18.00 | (ATG)_7_(ATT)_7_ | F: CTCCGGCAAGAGTGAACATT  R: CGACGGAGTACTTCGCATTT | 148 |
| 80 | SSR304 | 7 | 37.56 | (CCA)_7_ | F: TCCTCCGGTTGTTACTCCAC  R: TTAGCACTTCCACCGATTCC | 186 |
| 81 | SSR565 | 7 | 44.20 | (GGGGAT)_3_(GAT)_3_(GAG)_2_(AAC)_3_(CAG)_7_ | F: GAGGATGATGAGAACTCGCC  R: TCAGAGGCTTCTGGGTCAGT | 236 |
| 82 | SSR45 | 7 | 60.00 | (AAT)_14_ | F: TGTATCCTGGTGGACCAATG  R: TCCAAGTATCAGGCACACCA | 246 |
| 83 | SSR344 | 8 | 0.00 | (AT)_12_ | F: TGTTGCTCGAACTCTCCAAA  R: CATAGGAGAGGTAACCCGCA | 275 |
| 84 | SSR244 | 8 | 7.00 | (TA)_4_ | F: GCGATGGTCTGAGACACTGA  R: CAGCTGGTGATCCTCCTCTT | 201 |
| 85 | TOM168 | 8 | 8.00 | NA | F: GTAATAATAGTGCGGACAGATAA  R: AAGGTGGCTAATAAAAGAATGAT | NA |
| 86 | SSR327 | 8 | 22.50 | (AAT)_7_ | F: TCAGGATCAGGAGCAGGAGT  R: TGGACTTGTTCCATGAACCC | 149 |
| 87 | SSR15 | 8 | 22.70 | (CT)_12_ | F: CACTTGCCATCTTCTAGCCC  R: ATGGATGCCCAAATTGAAGA | 192 |
| 88 | SSR335 | 8 | 49.70 | (TCT)_6_ | F: CCTCTCCATTCTGTGGTGGT  R: AACCGTCCTCGATTTCACAC | 225 |
| 89 | SSR63 | 8 | 54.30 | (AT)_39_ | F: CCACAAACAATTCCATCTCA  R: GCTTCCGCCATACTGATACG | 250 |
| 90 | SSR38 | 8 | 55.00 | (TCT)_6_ | F: GTTTCTATAGCTGAAACTCAACCTG  R: GGGTTCATCAAATCTACCATCA | 237 |
| 91 | SSR594 | 8 | 58.00 | (TCT)_8_ | F: TTCGTTGAAGAAGATGATGGTC  R: CAAAGAGAACAAGCATCCAAGA | 293 |
| 92 | SSRB105694 | 8 | 87.00 | NA | F:AAAGCCAAAGTGGAAGAACTCAAGG  R: CTCGTAAAACGTTCATCAATCTCGC | NA |
| 93 | SSR340 | 9 | 18.00 | (GTTGA)2(GA)_7_ | F: TTCTCTCTGTCGCCATTGTG  R: AAATCAACGCCAATGGTAGG | 281 |
| 94 | Cosi52 | 9 | 30.00 | NA | F: GCCTTTCTTCCAGGATGCTA  R: CCCATTTTCCTTCTTCCTAGA | NA |
| 95 | SSR73 | 9 | 32.00 | (AG)_2_(AGA)_7_(TAGTGA)_2_ | F: TGGGAAGATCCTGATGATGG  R: TTCCCTTTCCTCTGGACTCA | 198 |
| 96 | SSR69 | 9 | 37.00 | (TAT)_7_ | F: TTGGCTGGATTATTCCTGTTG  R: GCATTTGATAGAAGGCCAGC | 127 |
| 97 | SSR70 | 9 | 42.00 | (AT)_20_ | F: TTTAGGGTGTCTGTGGGTCC  R: GGAGTGCGCAGAGGATAGAG | 120 |
| 98 | SSR99 | 9 | 50.35 | (AG)6(AT)_5_ | F: GCCTCGGATTCAATAGCATTA  R: CACAAAGAAGCAAACAACTCCA | 176 |
| 99 | SSRB56555 | 9 | 50.40 | NA | F: TGCGGTAGACCCACTGAGTTATCTC  R: AAAAGGGAAGCATTTCTTCTCTCCC | NA |
| 100 | SSR28 | 9 | 50.45 | (CT)_13_ | F: ACCAAATGGAAATGGGTCAA  R: CCCTAAGACTAACGACAACCAA | 164 |
| 101 | SSR237 | 9 | 50.37 | (AT)_11_ | F: GTGGTAACGGCAAAGGGACT  R: CTTATGGCCTTAGCAGCCAG | 178 |
| 102 | SSR19 | 9 | 51.00 | (AT)_16_ | F: CCGTTACCTTGGTCCATCAC  R: GGGAGATGCCACATCACATA | 188 |
| 103 | SSR383 | 9 | 57.30 | (AT)11 | F:ATTGTACAAAGACCCGTGGC  R:GTTGCACACTGGATCAATGC | 188 |
| 104 | LEOH 170 | 9 | 50 | NA | F:GGATTAGAAGAGAAAAACAAAAGCA  R:AGCCTTCTCAAATTCCTCCTC | NA |
| 105 | SSR110 | 9 | 61.00 | (AT)_10_ | F: TGTAACGTCAAACTTCAGGTG  R: CTCCGCAATGTGTTGTATGG | 188 |
| 106 | SSR112 | 9 | 80.00 | (AAT)_7_ | F: GGAACACAACCAAGAAGTGGA  R: TATCGGCTTAGGGTTGTTGG | 177 |
| 107 | SSR599 | 9 | 103.00 | (TCA)_6_(TCATTA)_2_ | F: GGATTTCTCATGGAGAATCAGTC  R: TCCCTTGATCTTGATGATGTTG | 286 |
| 108 | SSR333 | 9 | 109.00 | (TA)_13_ | F: GTTCCCGCTTGAGAAACAAC  R: CCAATGCTGGGACAGAAGAT | 204 |
| 109 | SSR4 | 10 | 3.00 | (CGG)_7_ | F: TTCTTCGGAGACGAAGGGTA  R: CCTTCAATCCTCCAGATCCA | 166 |
| 110 | SSR34 | 10 | 25.30 | (GA)_7_(TCGA)_2_ | F: TTCGGATAAAGCAATCCACC  R: TCGATTGTGTACCAACGTCC | 184 |
| 111 | SSR596 | 10 | 25.70 | (GA)_7_(TCGA)_2_ | F: TTCGGATAAAGCAATCCACC  R: TCGATTGTGTACCAACGTCC | 184 |
| 112 | SSR318 | 10 | 34.50 | (TA)_21_ | F: GCAGAGGATATTGCATTCGC  R: CAAACCGAACTCATCAAGGG | 279 |
| 113 | SSR248 | 10 | 35.0 | (TA)_21_ | F: GCATTCGCTGTAGCTCGTTT  R: GGGAGCTTCATCATAGTAACG | 249 |
| 114 | SR2159 | 10 | 46.00 | (CAA)_9_(CTTC)_2_ | F: ACAACAAAGGCAGCTGGTTC  R: GCTTCCGACAGCCCATATAA | 212 |
| 115 | SSR85 | 10 | 55.00 | (TAA)_7_ | F: ATCCGTTAGCTATTGTGCCG  R: TTGCCATGCACTTATCTTCG | 179 |
| 116 | SL10386i* | 10 | 70.80 | NA | F:TGGAGTTCTGGGTCACTTTG  R:ATAGCCATCCCAAACCACAC | NA |
| 117 | SSR223 | 10 | 75.00 | (TCT)_7_ | F: TGGCTGCCTCTTCTCTGTTT  R: TTTCTTGAAGGGTCTTTCCC | 191 |
| 118 | M13938 | 10 | 76.20 | NA | F:GAGTCAAAGTTTGCTCACATC  R:CTCTTCTGAACTTGCTTTGAG | NA |
| 129 | SSR479 | 10 | 86.00 | (GAGCGA)_2_(GC)_7_ | F: TGTAAGAGTGTCTGCCTGCAC  R: ATGGGTTCGGGTTAGCTCTT | 235 |
| 120 | SSR80 | 11 | 20.00 | (CAA)_7_ | F: GGCAAATGTCAAAGGATTGG  R: AGGGTCATGTTCTTGATTGTCA | 186 |
| 121 | SSR136 | 11 | 11.00 | (CAG)_7_ | F: GAAACCGCCTCTTTCACTTG  R: CAGCAATGATTCCAGCGATA | 148 |
| 122 | SSR67 | 11 | 24.00 | (AGA)2(AAG)_7_ | F: GCACGAGACCAAGCAGATTA  R: GGGCCTTTCCTCCAGTAGAC | 100 |
| 123 | SSR76 | 11 | 37.50 | (CGG)_7_ | F: ACGGGTCGTCTTTGAAACAA  R: CCACCGGATTCTTCTTCGTA | 148 |
| 124 | SSR46 | 11 | 40.00 | (AT)_14_ | F: CCGAGGCGAATCTTGAATAC  R: GCACCATCTCTTGTGCCTCT | 163 |
| 125 | TMS17 | 11 | 40.20 | NA | F: AATGTAACAACGTGTCATGATTC  R: AAGTCACAAACTAAGTTAGGG | NA |
| 126 | SL10615i* | 11 | 57.30 | NA | F:TTTCCACAGGTCATTCTTCC  R:TGGGGGATGAGAGTTGTAATG | NA |
| 127 | TOO28 | 12 | 33.00 | NA | F: AAAACTCCGGCAGAACTCC  R: GTTTCTTCTTCTTCCTCATCCCC | NA |
| 128 | TOM176 | 12 | 41.00 | NA | F: TCTCGTTCTCCATCAAATACAAT  R: CAACCATCAGCATAGAAACAT | NA |
| 129 | SSR345 | 12 | 42.22 | (TCC)_7_ | F: AAGCCAAGCTCGAACCTGTA  R: ATCCATGCTGTCGCTTTCAT | 179 |
| 130 | TG565 | 12 | 48.00 | NA | F: ATTCACACCCTGAAGCCTTG  R: TCGGAAACAGCCTCTCTACC | NA |
| 131 | TG111 | 12 | 55.00 | NA | F: GGGTTCGATACACCCTTAGC  R: CCTTTCTATATAATCCACCAAATGC | NA |
| 132 | SSR20 | 12 | 58.20 | (GAA)_8_ | F: GAGGACGACAACAACAACGA  R: GACATGCCACTTAGATCCACAA | 157 |
| 133 | SSR124 | 12 | 60.00 | (CACC)_2_(GA)_7_ | F: TCAATCCATCACACCTTGGA  R: GAGGAAGAAGACCACGCAAA | 131 |
| 134 | SSR44 | 12 | 60.00 | (GA)_54_ | F: TCATCTGCAATTCATGGCTC  R: AGGTCAAGGATGTGCTTCCC | 220 |
| 135 | Leoh301* | 12 | 68.00 | NA | F: TCTGATGTTGGTAGAGCCATTG  R: TGATCATAATGTGACGAATCGAA | NA |

**NA.** Not available. **_*_.** Indel markers
