# Supplementary material for: Nitric Oxide Overproduction in Tomato shr Mutant Shifts Metabolic Profiles and Suppresses Fruit Growth and Ripening
Source: Front Plant Sci. 2016 Nov 28;7:1714. doi: 10.3389/fpls.2016.01714 (PMC5124567; doi:10.3389/fpls.2016.01714)
Supplement: Supplementary Table S2 — List of additional Simple Sequence Repeats (SSR) markers selected from Kazusa DNA research institute (http://marker.kazusa.or.jp/tomato). [file Table2.DOCX]

**Supplementary Material**

**Nitric oxide overproduction in tomato shr mutant alters cellular homeostasis and suppresses fruit growth and ripening**

*Reddaiah Bodanapu, Suresh Kumar Gupta, Pinjari Osman Basha, Kannabiran Sakthivel, Sadhna, Yellamaraju Sreelakshmi and Rameshwar Sharma*

**Corresponding author:** rameshwar.sharma@gmail.com

**Table S2.** List of additional Simple Sequence Repeats (SSR) markers selected from Kazusa DNA research institute (<http://marker.kazusa.or.jp/tomato>).

| **S. No** | **Marker name** | **Chromosome** | **Position**  **(cM)** | **Repeat type and length** | **Primer sequence (5'→3')**  **(F: Forward primer, R: Reverse primer)** | **Product size (bp)** |
| --- | --- | --- | --- | --- | --- | --- |
| 1 | TES1473 | 9 | 50.98 | (AAG)_6_ | F:GCACGAACCATACAGCTTCA  R:TGCTAACAGAAATGCTAGCACAA | 191 |
| 2 | TES1123 | 9 | 51.65 | (AAG)_7_ | F:AAAAGGTTTCCACATAAACCCA  R:GAGTTGTGGAGGGGAATTGA | 171 |
| 3 | TGS0906 | 9 | 52.47 | (AT)_12_ | F:TACACTTCTGGGCCCTATCG  R:GTCCAATTCCAAGCCAGAA | 202 |
| 4 | TGS1180 | 9 | 53.38 | (AT)_11_ | F: GTCTAAGATGGCAAAAGGGACA  R: CATGGCGATGTGGTGAATTA | 287 |
| 5 | TGS0213 | 9 | 54.17 | (AT)_25_ | F: GTGGGATAGTTGCGGAAAAA  R: AGCACGAATGTCCAATGCTAA | 168 |
| 6 | TGS2114 | 9 | 55.04 | (AAT)_7_ | F: GAAGATTCACGTCAGGGGTCA  R: AGATCACTGGCTCACTGCAA | 163 |
| 7 | TGS0781 | 9 | 55.38 | (AAT)_9_ | F:GTTTGCCGTAATTTTTGCCT  R:AGGTTGATACTTGAGAGATATAGCAACA | 263 |
| 8 | TGS3371 | 9 | 55.83 | (AAG)_2_ | F: TGATCGAACACGTTGAAAGC  R: GATCGAAATGGAGACGGAAA | 224 |
| 9 | TGS0470 | 9 | 56.50 | (AT)_18_ | F: GATATGACCCTTTGCCACTGC  R: CAATATGAAATCGAGGGAGCA | 186 |
| 10 | TES0840 | 9 | 56.82 | ATC | F:GTAAACCCCAATTCCCTACCC R:CGTCTCAGTCACCGATGTTG | 253 |
| 11 | TGS1268 | 9 | 57.37 | (AAT)_7_ | F:TCCAAACAATTGTATCGTTCTCA  R: GTCCTACCCTCCCCAGACTC | 104 |
| 12 | TGS3324 | 9 | 58.26 | (GGT)_5_ | F: GAGTAGAAGGGGGAGGAGGA  R: CCAATTTGTTTCTTCGCACA | 295 |
| 13 | TGS1365 | 9 | 59.51 | (AAAT)_5_ | F: GCATGAATGGGTGGTTCATCA  R: ACCCCTAGCTAGCTCCAACC | 206 |
| 14 | TGS0801 | 9 | 59.84 | (AAT)_9_ | F:GCTGGGCAAGAAATTTCGGTA  R:TGGTCTCTTCCGAGCCTCTA | 235 |
