# Supplementary material for: Nitric Oxide Overproduction in Tomato shr Mutant Shifts Metabolic Profiles and Suppresses Fruit Growth and Ripening
Source: Front Plant Sci. 2016 Nov 28;7:1714. doi: 10.3389/fpls.2016.01714 (PMC5124567; doi:10.3389/fpls.2016.01714)
Supplement: Supplementary Table S3 — List of additional Simple Sequence Repeats (SSR) markers selected from Veg Marks, a DNA marker database for vegetables (http://vegmarks.nivot.affrc.go.jp, Last accessed in 2013). [file Table3.DOCX]

**Supplementary Material**

**Nitric oxide overproduction in tomato shr mutant alters cellular homeostasis and suppresses fruit growth and ripening**

*Reddaiah Bodanapu, Suresh Kumar Gupta, Pinjari Osman Basha, Kannabiran Sakthivel, Sadhna, Yellamaraju Sreelakshmi and Rameshwar Sharma*

**Corresponding author:** rameshwar.sharma@gmail.com

**Table S3.** List of additional Simple Sequence Repeats (SSR) markers selected from Veg Marks, a DNA marker database for vegetables (<http://vegmarks.nivot.affrc.go.jp>).

| **S. No** | **Marker name** | **Chromosome** | **Position**  **(cM)** | **Repeat type and length** | **Primer sequence (5'→3')**  **(F: Forward primer, R: Reverse primer)** | **Product size (bp)** |
| --- | --- | --- | --- | --- | --- | --- |
| 1 | TMA0067 | 9 | 51.67 | (TA)_4_TCTAGAAAATAT(TA)_4_ | F: TGGAGTTTTTGAGCTTCATCTCTGA  R: AGCGCCCTAATTTTCCCTATGAAT | 299 |
| 2 | TMA0224 | 9 | 51.67 | (TA)_20_ | F: CTGCATCTCCGATTTGAGCTTTTT  R: TTTCTTCGAGTACCTCCTTGGTGC | 191 |
| 3 | TMB0082 | 9 | 51.67 | (TA)_3_T(AC)_13_TTTTTCGTAGCTTAAATTTAATTTTA(AT)_3_ | F: CTGGGGTGTTATGGCCAAATAGAG  R: CCTAATTCTTCTTTGGGGTCTTGC | 215 |
| 4 | TMB0128 | 9 | 52.40 | (AG)_11_ | F:GGGGTTGGAGTTATTCTAATCCTAGAC  R: ATGGGCATAAGCCCTCGTTAATTT | 241 |
| 5 | TME0014 | 9 | 53.93 | (TA)_7_ | F: GTCCGTCATCAACCAGTCAGCAG  R: CCAATGGACGGATCATTGCTATTAC | 271 |
| 6 | TMA0144 | 9 | 57.23 | (TA)_23_TG(TA)_5_  TG(TA)_3_ | F: CAAGTATGCGAGCTCAATTGCTAA  R: CTTAGAGAATCTCCCACAGAAACAGT | 300 |
| 7 | Tmb0006 | 9 | 61.34 | (AG)_17_GAA(AT)_3_GAGCTT(GA)_3_CTTGAAATTTGGAT(GA)_3_ | F: AGAGAAGGTGATCTTGTGGCTGCT  R: AAAATGAACTACAGGAGAGGGGGC | 249 |
| 8 | TMA0288 | 9 | 66.72 | (TA)_15_T(AC)_3_A(AC)_5_ | F: CCACTGCCACCATTTAATGCTTTT  R: TGTCAATATGAAATCGAGGGAGCA | 176 |
| 9 | TME0213 | 9 | 71.86 | (AC)_5_AAACAACCCAAA(AC)_4_TTCCATTTCCATTTTTTGCC  (TA)_9_ | F: TCTTCATGGCTCTCACTGCTCTTT  R: CAATCTTTCTTGGATCGGAGCTGT | 252 |
| 10 | TMA0228 | 9 | 74.76 | (GAA)_3_A(TA)_20_ | F: GTCATTCAAGATGAAAGGGTAAAGG  R: TGCAGGAATACACTGGTCGTTCTG | 252 |
