# Supplementary material for: Nitric Oxide Overproduction in Tomato shr Mutant Shifts Metabolic Profiles and Suppresses Fruit Growth and Ripening
Source: Front Plant Sci. 2016 Nov 28;7:1714. doi: 10.3389/fpls.2016.01714 (PMC5124567; doi:10.3389/fpls.2016.01714)
Supplement: Supplementary Table S4 — List of Cleaved Amplified Polymorphic Sequences (CAPS) markers used to map shr locus on the chromosome (http://solgenomics.net). [file Table4.DOCX]

**Supplementary Material**

**Nitric oxide overproduction in tomato shr mutant alters cellular homeostasis and suppresses fruit growth and ripening**

*Reddaiah Bodanapu, Suresh Kumar Gupta, Pinjari Osman Basha, Kannabiran Sakthivel, Sadhna, Yellamaraju Sreelakshmi and Rameshwar Sharma*

**Corresponding author:** rameshwar.sharma@gmail.com

**Table S4.** List of Cleaved Amplified Polymorphic Sequences (CAPS) markers used to map *shr* locus on the chromosome (<http://solgenomics.net>).

| **S. No** | **Marker name** | **Chromosome** | **Position**  **(cM)** | **Repeat type and length** | **Primer sequence (5'→3')**  **(F: Forward primer, R: Reverse primer)** | **Product size (bp)** |
| --- | --- | --- | --- | --- | --- | --- |
| 1 | [C2_At3g63190](http://solgenomics.net/search/markers/markerinfo.pl?marker_id=8127) | 09 | 52.00 | Hinf1 | F: TTGGTGCAGCCGTATGACAAATCC  R: TCCATCATTATTTGGCGTCATACC | 500 |
| 2 | [C2_At4g02580](http://solgenomics.net/search/markers/markerinfo.pl?marker_id=8168) | 09 | 52.00 | Hinf1 | F: AACAAAACGGACCTTCCTTGGGAG  R: AGTTGCAACCTCATATACACGAATTGG | 1800 |
| 3 | [C2_At2g29210](http://solgenomics.net/search/markers/markerinfo.pl?marker_id=7348) | 09 | 55.00 | Apo1 | F: AGCAGGACACTCGATTCTCTAATAAGC  R: TGCACTAAGTAGTAATGCCCAAAGCTC | 450 |
| 4 | [C2_At4g02680](http://solgenomics.net/search/markers/markerinfo.pl?marker_id=8170) | 09 | 56.30 | Hinf1 | F: ATGAGGAGTTTACAGTTGGCTCGC  R: TCTGATCTCTTCTCGTAGGCAGATGC | 900 |
| 5 | [C2_At1g02910](http://solgenomics.net/search/markers/markerinfo.pl?marker_id=6483) | 09 | 57.70 | Dra1 | F: TGAACCCACTCCCACTGCTGAGTC  R: TGCTGTGTCGAATAGCACAAGAGC | 1600 |
| 6 | [C2_At4g03200](http://solgenomics.net/search/markers/markerinfo.pl?marker_id=8181) | 09 | 58.00 | Msp1 | F: AGAAAGCAACATAGGTTATTAGGAAAC  R: TCACAGAAACGGAATTCCCTGAAGG | 400 |
| 7 | [U228448](http://solgenomics.net/search/markers/markerinfo.pl?marker_id=9544) | 09 | 59.50 | Dra1 | F: TGAACCCACTCCCACTGCTGAGTC  R: TGCTGTGTCGAATAGCACAAGAGC | 1600 |
