# Supplementary material for: Nitric Oxide Overproduction in Tomato shr Mutant Shifts Metabolic Profiles and Suppresses Fruit Growth and Ripening
Source: Front Plant Sci. 2016 Nov 28;7:1714. doi: 10.3389/fpls.2016.01714 (PMC5124567; doi:10.3389/fpls.2016.01714)
Supplement: Supplementary Table S5 — The genetic segregation of short root phenotype. The segregation was analyzed in the progeny of shr x S. pimpinellifolium and WT x shr. The seedlings were grown under white light and segregation of short root, and long root phenotype in F1 and F2 generation was analyzed 7–9 days after germination. [file Table5.DOCX]

**Supplementary Material**

**Nitric oxide overproduction in tomato shr mutant alters cellular homeostasis and suppresses fruit growth and ripening**

*Reddaiah Bodanapu, Suresh Kumar Gupta, Pinjari Osman Basha, Kannabiran Sakthivel, Sadhna, Yellamaraju Sreelakshmi and Rameshwar Sharma*

**Corresponding author:** rameshwar.sharma@gmail.com

**Table S5:** The genetic segregation of short root phenotype. The segregation was analyzed in the progeny of *shr* x *S. pimpinellifolium* and WT x *shr*. The seedlings were grown under white light and segregation of short root, and long root phenotype in F_1_ and F_2_ generation was analyzed 7-9 days after germination.

| **Cross** | **Number of Plants** | | | | | |
| --- | --- | --- | --- | --- | --- | --- |
|  | **F_1_ generation** | | **F_2_ generation** | | | |
|  | **Short roots** | **Long roots** | **Short roots** | **Long roots** | **χ2 (3:1, df 1)** | **P** |
| *shr* x *S. pimpinellifolium* | 0 | 9 | 192 | 577 | 0.0013 | 0.983 |
| WT x *shr* (Negi et al. 2010) |  |  | 81 | 241 |  | 0.99 |
