# Supplementary material for: Nitric Oxide Overproduction in Tomato shr Mutant Shifts Metabolic Profiles and Suppresses Fruit Growth and Ripening
Source: Front Plant Sci. 2016 Nov 28;7:1714. doi: 10.3389/fpls.2016.01714 (PMC5124567; doi:10.3389/fpls.2016.01714)
Supplement: Supplementary Table S6 — The segregation of lateral root phenotype in the progeny of shr x S. pimpinellifolium. The seedlings were grown under white light and segregation of lateral root, and no lateral root phenotype in F1 and F2 generation was analyzed 7–9 days after germination. The identical segregation ratio for lateral root was obtained for another tomato mutant in Ailsa Craig background that was crossed with S. pimpinellifolium (data not shown) indicating that lateral root gene was contributed by S. lycopersicon and was unrelated to shr locus. [file Table6.DOCX]

**Supplementary Material**

**Nitric oxide overproduction in tomato shr mutant alters cellular homeostasis and suppresses fruit growth and ripening**

*Reddaiah Bodanapu, Suresh Kumar Gupta, Pinjari Osman Basha, Kannabiran Sakthivel, Sadhna, Yellamaraju Sreelakshmi and Rameshwar Sharma*

**Corresponding author:** rameshwar.sharma@gmail.com

**Table S6:** The segregation of lateral root phenotype in the progeny of *shr* x *S. pimpinellifolium*. The seedlings were grown under white light and segregation of lateral root, and no lateral root phenotype in F_1_ and F_2_ generation was analyzed 7-9 days after germination. The identical segregation ratio for lateral root was obtained for another tomato mutant in Ailsa Craig background that was crossed with *S. pimpinellifolium* (data not shown) indicating that lateral root gene was contributed by *S. lycopersicon* and was unrelated to *shr* locus.

| **Cross** | **Number of Plants** | | | | | | |
| --- | --- | --- | --- | --- | --- | --- | --- |
|  | **F_1_ generation** | | **F_2_ generation** | | | | |
| *shr* X *S. pimpinellifolium* | **No lateral roots** | **Lateral  roots** | **No lateral roots** | **Lateral  roots** | **Ratio tested** | **χ2** | P |
|  | 0 | 17 | 41 | 89 | 3:1 | 2.964 | 0.0851 |
