# Supplementary material for: Nitric Oxide Overproduction in Tomato shr Mutant Shifts Metabolic Profiles and Suppresses Fruit Growth and Ripening
Source: Front Plant Sci. 2016 Nov 28;7:1714. doi: 10.3389/fpls.2016.01714 (PMC5124567; doi:10.3389/fpls.2016.01714)
Supplement: Supplementary Table S7 — Genotype frequency for molecular markers on chromosome 9 in the mapping population derived from shr x S. pimpinellifolium. [file Table7.DOCX]

**Supplementary Material**

**Nitric oxide overproduction in tomato shr mutant alters cellular homeostasis and suppresses fruit growth and ripening**

*Reddaiah Bodanapu, Suresh Kumar Gupta, Pinjari Osman Basha, Kannabiran Sakthivel, Sadhna, Yellamaraju Sreelakshmi and Rameshwar Sharma*

**Corresponding author:** rameshwar.sharma@gmail.com

**Table S7**: Genotype frequency for molecular markers on chromosome 9 in the mapping population derived from *shr* x *S. pimpinellifolium*.

| **Marker name** | **F_2_ genotype** | | | | **χ2**  **(1:2:1, df 2)** | **P** |
| --- | --- | --- | --- | --- | --- | --- |
|  | Homozygous for *shr* | Heterozygous | Homozygous for *S. pimpinellifolium* | Missing data |  |  |
| SSR19 | 188 | 362 | 195 | 24 | 0.717 | 0.696 |
| SSR110 | 172 | 388 | 184 | 25 | 1.76 | 0.414 |
| SSR383 | 187 | 377 | 176 | 29 | 0.59 | 0.743 |
| C2_At3g63190 | 195 | 396 | 162 | 16 | 4.894 | 0.085 |
| TGS0213 | 197 | 366 | 191 | 15 | 0.736 | 0.68 |
