# Supplementary material for: Nitric Oxide Overproduction in Tomato shr Mutant Shifts Metabolic Profiles and Suppresses Fruit Growth and Ripening
Source: Front Plant Sci. 2016 Nov 28;7:1714. doi: 10.3389/fpls.2016.01714 (PMC5124567; doi:10.3389/fpls.2016.01714)
Supplement: Supplementary Table S8 — List of genes between SSR marker TGS0213 and CAPS marker C2_At3g6310 (http://solgenomics.net/gb2/gbrowse/ITAG2.3_genomic/). Expression of genes was retrieved from Tomato Genome Consortium (2012). [file Table8.PDF]

Supplementary Table S8. List of genes between SSR marker TGS0213 and CAPS marker C2_At3g6310 (http://solgenomics.net/gb2/gbrowse/ITAG2.3_genomic/). Expression of genes was retrieved from Tomato Genome Consortium (2012).

S.No	SGN ID	Gene Name	Root
1	Solyc09g061260	Calmodulin protein kinase (Fragment) (AHRD V1 **-- D3K3H9_LONJA)	0
2	Solyc09g061270	BSD domain-containing protein (AHRD V1 ***- D7L5T3_ARALY); contains Interpro domain(s) IPR005607	11.27
3	Solyc09g061280	Cyclin dependent kinase inhibitor (AHRD V1 **** Q5PXG5_EUPES); contains Interpro domain(s) IPR0167	10.99
4	Solyc09g061290	WD-40 repeat family protein (AHRD V1 **-- D7LF16_ARALY); contains Interpro domain(s) IPR017986 W	45.125
5	Solyc09g061300	Unknown Protein (AHRD V1)	0
6	Solyc09g061310	PPPDE peptidase domain containing 2a (AHRD V1 *--- Q6PC81_DANRE); contains Interpro domain(s) IPR	47.09
7	Solyc09g061320	Solute carrier family 35 member F4 (AHRD V1 **-- C0HBH8_SALSA); contains Interpro domain(s) IPR000	8.9
8	Solyc09g061330	Serine/threonine-protein kinase (AHRD V1 **** C6ZRV0_SOYBN); contains Interpro domain(s) IPR0022	0.115
9	Solyc09g061340	Pentatricopeptide repeat-containing protein (AHRD V1 ***- D7L041_ARALY); contains Interpro domain(s)	2.95
10	Solyc09g061350	Unknown Protein (AHRD V1)	0
11	Solyc09g061360	Calmodulin protein kinase (Fragment) (AHRD V1 ***- D3K3H9_LONJA)	0
12	Solyc09g061370	Protein VERNALIZATION insensitive 3 (AHRD V1 *--- D7MN96_ARALY)	0
13	Solyc09g061380	NHL repeat (AHRD V1 *-*- Q3M6W0_ANAVT); contains Interpro domain(s) IPR011042 Six-bladed beta-p	8.78
14	Solyc09g061390	Maturase K (AHRD V1 ***- B0LBN3_9SOLA); contains Interpro domain(s) IPR002866 Maturase, MatK, N	1.035
15	Solyc09g061400	Ulp1 protease family C-terminal catalytic domain containing protein (AHRD V1 ***- Q60D46_SOLDE); cont	0.13
16	Solyc09g061410	Unknown Protein (AHRD V1)	18.355
17	Solyc09g061420	Os02g0515000 protein (Fragment) (AHRD V1 *-*- Q0E0X2_ORYSJ)	4.805
18	Solyc09g061430	Nucleolar GTP-binding protein 2 (AHRD V1 *-** B0WF29_CULQU); contains Interpro domain(s) IPR0148	29.89
19	Solyc09g061440	Os04g0513000 protein (Fragment) (AHRD V1 *-*- Q0JBS2_ORYSJ)	27.86
20	Solyc09g061450	30S ribosomal protein S7 chloroplastic (AHRD V1 *-*- D3WBE1_9BORA); contains Interpro domain(s) IPR	3.36
21	Solyc09g061460	NAD(P)H-quinone oxidoreductase subunit 2 chloroplastic (AHRD V1 ***- D2KLR3_OLEEU); contains Inte	0.06
22	Solyc09g061470	Unknown Protein (AHRD V1)	0
23	Solyc09g061480	Unknown Protein (AHRD V1)	0
24	Solyc09g061490	Calmodulin protein kinase (Fragment) (AHRD V1 ***- D3K3H9_LONJA)	0
25	Solyc09g061500	Calmodulin protein kinase (Fragment) (AHRD V1 ***- D3K3H9_LONJA)	0
26	Solyc09g061510	Unknown Protein (AHRD V1)	0
27	Solyc09g061520	Unknown Protein (AHRD V1)	0
28	Solyc09g061530	Unknown Protein (AHRD V1)	0
29	Solyc09g061540	Calmodulin protein kinase (Fragment) (AHRD V1 ***- D3K3H9_LONJA)	0
30	Solyc09g061550	Receptor like protein kinase (AHRD V1 ***- Q39139_ARATH); contains Interpro domain(s) IPR000719 Pr	0
31	Solyc09g061560	Calmodulin protein kinase (Fragment) (AHRD V1 ***- D3K3H9_LONJA)	0
32	Solyc09g061570	Receptor like protein kinase (AHRD V1 ***- Q39139_ARATH); contains Interpro domain(s) IPR000719 Pr	0
33	Solyc09g061580	Calmodulin protein kinase (Fragment) (AHRD V1 ***- D3K3H9_LONJA)	0
34	Solyc09g061590	Calmodulin protein kinase (Fragment) (AHRD V1 ***- D3K3H9_LONJA)	0
35	Solyc09g061600	Calmodulin protein kinase (Fragment) (AHRD V1 ***- D3K3H9_LONJA)	0
36	Solyc09g061610	Unknown Protein (AHRD V1); contains Interpro domain(s) IPR001209 Ribosomal protein S14	0.165
37	Solyc09g061620	Vesicle-associated membrane protein 7B (AHRD V1 ***- D7KE73_ARALY); contains Interpro domain(s) IP	44.435
38	Solyc09g061630	Unknown Protein (AHRD V1)	0.75
39	Solyc09g061640	SWIM zinc finger family protein (AHRD V1 *--- Q2R366_ORYSJ); contains Interpro domain(s) IPR006564	0.09
40	Solyc09g061650	Serine/threonine-protein phosphatase 7 long form homolog (AHRD V1 ***- PPP7L_ARATH); contains Interp	1.73
41	Solyc09g061660	Serine/threonine-protein phosphatase 7 long form homolog (AHRD V1 *--- PPP7L_ARATH); contains Interpr	0.245
42	Solyc09g061670	Serine/threonine-protein phosphatase 7 long form homolog (AHRD V1 **-- PPP7L_ARATH); contains Interp	0.075
43	Solyc09g061680	Pantothenate synthetase (AHRD V1 ***- D2C4V4_THENR); contains Interpro domain(s) IPR003721 Panto	14.01
44	Solyc09g061690	Serine/threonine-protein phosphatase 7 long form homolog (AHRD V1 **-- PPP7L_ARATH); contains Interp	1.49
45	Solyc09g061700	Male sterility 5 family protein (Fragment) (AHRD V1 *--- B1Q393_BRAOT); contains Interpro domain(s) IP	1.295
46	Solyc09g061710	Ribonuclease P protein subunit p25 (AHRD V1 *--- RPP25_HUMAN); contains Interpro domain(s) IPR0027	12.735
47	Solyc09g061720	Unknown Protein (AHRD V1)	21.315
48	Solyc09g061730	Genomic DNA chromosome 3 TAC clone K7P8 (AHRD V1 ***- Q9LRY4_ARATH)	11.315
49	Solyc09g061750	Os07g0419800 protein (Fragment) (AHRD V1 *--- Q0D6Y1_ORYSJ); contains Interpro domain(s) IPR0128	26.025
50	Solyc09g061760	Pentatricopeptide repeat-containing protein (AHRD V1 ***- D7KS33_ARALY); contains Interpro domain(s)	0.625
51	Solyc09g061770	cDNA clone J013073D14 full insert sequence (AHRD V1 *--- B7ECD3_ORYSJ); contains Interpro domain(s	1.325
52	Solyc09g061780	Os07g0419800 protein (Fragment) (AHRD V1 *--- Q0D6Y1_ORYSJ); contains Interpro domain(s) IPR0128	0
53	Solyc09g061790	Pentatricopeptide repeat (AHRD V1 ***- A2Q513_MEDTR); contains Interpro domain(s) IPR002885 Penta	0.42
54	Solyc09g061820	Unknown Protein (AHRD V1)	0
55	Solyc09g061830	Unknown Protein (AHRD V1)	0.465
56	Solyc09g061840	3-ketoacyl CoA thiolase 1 (AHRD V1 **** C8YNG6_PETHY); contains Interpro domain(s) IPR002155 Thi	36.675
57	Solyc09g061850	Protein FAR1-RELATED SEQUENCE 5 (AHRD V1 **-- FRS5_ARATH); contains Interpro domain(s) IPR0	0
58	Solyc09g061860	Sterol 3-beta-glucosyltransferase (AHRD V1 ***- B6U4Q7_MAIZE); contains Interpro domain(s) IPR00427	16.03
59	Solyc09g061870	Unknown Protein (AHRD V1)	0
60	Solyc09g061880	Unknown Protein (AHRD V1)	0.37
61	Solyc09g061890	Pectate lyase 1-27 (AHRD V1 **** B9TU35_9ROSI); contains Interpro domain(s) IPR002022 Pectate lyase	13.515
62	Solyc09g061900	Unknown Protein (AHRD V1)	0
63	Solyc09g061910	Unknown Protein (AHRD V1)	0
64	Solyc09g061920	Unknown Protein (AHRD V1)	0
65	Solyc09g061930	Receptor like kinase, RLK	12.67
66	Solyc09g061940	Receptor like kinase, RLK	1.6
67	Solyc09g061950	MADS box transcription factor-like protein (AHRD V1 **** Q9FLH5_ARATH); contains Interpro domain(s)	0

68	Solyc09g061960	Late embryogenesis abundant protein (AHRD V1 ***- Q2HV86_MEDTR); contains Interpro domain(s) IPR(	0.245
		Gap region 35116 bp (SL2.40ch09:56426627...56461743)	
69	Solyc09g062960	Unknown Protein (AHRD V1)	0.14
70	Solyc09g062970	Unknown Protein (AHRD V1)	1.125
71	Solyc09g062980	Unknown Protein (AHRD V1)	0.155
72	Solyc09g062990	Unknown Protein (AHRD V1)	0
73	Solyc09g063000	Unknown Protein (AHRD V1)	0
74	Solyc09g063010	BHLH transcription factor (AHRD V1 *-** B6TXR4_MAIZE); contains Interpro domain(s) IPR011598 Hel	1.02
75	Solyc09g063020	Transport protein yif1 (AHRD V1 **-- C1H1D3_PARBA); contains Interpro domain(s) IPR005578 Hrf1	10.295
76	Solyc09g063030	CLIP-associating protein 1-like (AHRD V1 *--- Q6KAI5_ORYSJ); contains Interpro domain(s) IPR011989 A	32.47
77	Solyc09g063040	Nbs-lrr, resistance protein	0
78	Solyc09g063050	Pentatricopeptide repeat-containing protein (AHRD V1 ***- D7L3A6_ARALY); contains Interpro domain(s)	0
79	Solyc09g063060	GDSL esterase/lipase At4g28780 (AHRD V1 ***- GDL67_ARATH); contains Interpro domain(s) IPR00108'	0.08
80	Solyc09g063070	Senescence-associated protein-like (ISS) (AHRD V1 ***- Q00X72_OSTTA)	36.035
81	Solyc09g063080	Genomic DNA chromosome 5 BAC clone F6B6 (AHRD V1 ***- Q9LKC1_ARATH)	21.82
82	Solyc09g063090	Unknown Protein (AHRD V1)	16.525
83	Solyc09g063110	Ulp1 protease family C-terminal catalytic domain containing protein (AHRD V1 ***- Q60D46_SOLDE); cont	0
84	Solyc09g063120	Unknown Protein (AHRD V1)	0
85	Solyc09g063130	Photosystem I reaction center subunit IV A (AHRD V1 *-*- B6TH55_MAIZE); contains Interpro domain(s) I	3.08
86	Solyc09g063140	Os06g0235500 protein (Fragment) (AHRD V1 *-*- Q0DDB7_ORYSJ)	3.675
87	Solyc09g063150	Glutathione S-transferase (AHRD V1 **** Q8H9E5_CUCMA); contains Interpro domain(s) IPR017933 Glu	51.595
		Gap region 31568 bp (SL2.40ch09:56795954...56827522)	
88	Solyc09g064150	HAT family dimerisation domain containing protein (AHRD V1 ***- Q2R194_ORYSJ); contains Interpro doi	0
89	Solyc09g064160	Flavin monooxygenase-like protein (AHRD V1 **** D2IGV4_SOLLC); contains Interpro domain(s) IPR000	1.925
90	Solyc09g064170	Unknown Protein (AHRD V1)	29.935
91	Solyc09g064180	Unknown Protein (AHRD V1)	0.775
92	Solyc09g064190	Mutator-like transposase (AHRD V1 ***- Q9FW81_ORYSJ); contains Interpro domain(s) IPR006564 Zinc f	0.045
93	Solyc09g064200	Myosin XI-2 (AHRD V1 ***- B0CN59_NICBE); contains Interpro domain(s) IPR001609 Myosin head, mot	27.915
94	Solyc09g064220	HAT family dimerisation domain containing protein (AHRD V1 *-*- Q53M47_ORYSJ)	0
95	Solyc09g064230	Phosphoribosylanthranilate transferase (Fragment) (AHRD V1 *--- Q43085_PEA); contains Interpro domain(s	0.475
96	Solyc09g064240	Kinase pfkB family protein (AHRD V1 ***- Q7XDM4_ORYSJ); contains Interpro domain(s) IPR011611 Ca	10.79
97	Solyc09g064250	Unknown Protein (AHRD V1)	0
98	Solyc09g064260	Unknown Protein (AHRD V1)	0
99	Solyc09g064270	Receptor-like kinase (AHRD V1 **** A7VM33_MARPO); contains Interpro domain(s) IPR002290 Serine/t	20.705
100	Solyc09g064280	Unknown Protein (AHRD V1)	26.505
101	Solyc09g064290	Transposase (Fragment) (AHRD V1 *-*- Q0GJY7_9POAL); contains Interpro domain(s) IPR002559 Transp	0.025
102	Solyc09g064300	Zinc finger family protein (AHRD V1 ***- D7LUL3_ARALY)	0
103	Solyc09g064310	WD-repeat protein-like (AHRD V1 ***- Q9FND4_ARATH); contains Interpro domain(s) IPR019782 WD4(	3.2
104	Solyc09g064320	WD-repeat protein-like (AHRD V1 **-- Q9FND4_ARATH); contains Interpro domain(s) IPR017986 WD40	4.35
105	Solyc09g064330	Enzyme of the cupin superfamily (AHRD V1 ***- B6T5M3_MAIZE); contains Interpro domain(s) IPR00857	1.86
106	Solyc09g064340	Enzyme of the cupin superfamily (AHRD V1 ***- B6T5M3_MAIZE); contains Interpro domain(s) IPR00857	0.48
107	Solyc09g064350	Enzyme of the cupin superfamily (AHRD V1 ***- B6T5M3_MAIZE); contains Interpro domain(s) IPR01471	0.465
108	Solyc09g064360	Potassium channel tetramerization domain-containing protein (AHRD V1 *-*- D7LHH7_ARALY)	0
109	Solyc09g064370	Alcohol dehydrogenase (AHRD V1 **** Q1HQD0_BOMMO); contains Interpro domain(s) IPR014183 Alc	105.635
110	Solyc09g064380	TPR Domain containing protein expressed (AHRD V1 **-- Q10CT3_ORYSJ)	3.41
111	Solyc09g064390	Tetratricopeptide repeat protein 4 (AHRD V1 **-- B0WYL6_CULQU); contains Interpro domain(s) IPR011(	7.335
112	Solyc09g064400	Photosystem I reaction center subunit IX (AHRD V1 ***- Q06R45_9LAMI); contains Interpro domain(s) IPF	3.34
113	Solyc09g064410	50S ribosomal protein L33 chloroplastic (AHRD V1 ***- D3WBK4_9AQUA); contains Interpro domain(s) I	0.765
114	Solyc09g064420	LOC100158433 protein (Fragment) (AHRD V1 *-*- B1WBD4_XENLA)	8.035
115	Solyc09g064430	Aromatic L-amino acid decarboxylase (AHRD V1 **** A6BM84_ROSDA); contains Interpro domain(s) IPR	20.52
116	Solyc09g064440	ABC transporter C family member 5 (AHRD V1 **-- AB5C_ARATH); contains Interpro domain(s) IPR0011-	19.475
117	Solyc09g064450	NADH dehydrogenase (Ubiquinone) (AHRD V1 **** D7FUY2_ECTSI); contains Interpro domain(s) IPR01	99.48
118	Solyc09g064460	Unknown Protein (AHRD V1)	0.765
119	Solyc09g064470	Galactosyltransferase-like protein (Fragment) (AHRD V1 *--- D8SQH4_SELML); contains Interpro domain(s	26.64
120	Solyc09g064480	Galactosyltransferase-like protein (Fragment) (AHRD V1 **-- D8SQH4_SELML); contains Interpro domain(s	35.02
121	Solyc09g064490	Subtilisin-like protease (AHRD V1 **-- B6U0R8_MAIZE); contains Interpro domain(s) IPR015500 Peptidas	0
122	Solyc09g064500	Photosystem II reaction center psb28 protein (AHRD V1 ***- C6SZM2_SOYBN); contains Interpro domain(s	1.055
123	Solyc09g064510	Transcriptional activator TenA family (AHRD V1 *-*- Q117V2_TRIEI); contains Interpro domain(s) IPR004	27.495
124	Solyc09g064520	Receptor like kinase, RLK	9.23
125	Solyc09g064530	Auxin responsive protein (AHRD V1 *-*- B3U2A2_CUCSA); contains Interpro domain(s) IPR003311 AUX	13.3
126	Solyc09g064540	Genomic DNA chromosome 5 P1 clone MRH10 (AHRD V1 **-- Q9FND1_ARATH); contains Interpro doma	4.355
127	Solyc09g064550	Unknown Protein (AHRD V1)	0
128	Solyc09g064560	Unknown Protein (AHRD V1)	0
129	Solyc09g064570	Unknown Protein (AHRD V1)	0
130	Solyc09g064580	Photosystem II reaction center protein M (AHRD V1 *-*- Q45YZ7_9ERIC); contains Interpro domain(s) IPR	0.63
131	Solyc09g064590	Pre-mRNA-processing factor 39 (AHRD V1 *-** C5FSC0_NANOT); contains Interpro domain(s) IPR00310	31.975
132	Solyc09g064600	Lrr, resistance protein fragment	3.305
133	Solyc09g064610	Cc-nbs-lrr, resistance protein	7.335
134	Solyc09g064620	Unknown Protein (AHRD V1)	0.42
135	Solyc09g064630	TPR domain protein (AHRD V1 *--- B6U810_MAIZE); contains Interpro domain(s) IPR011990 Tetratricop(	55.08
136	Solyc09g064640	FAM65A protein related (AHRD V1 ***- Q6L3W5_SOLDE)	0

137	Solyc09g064650	Unknown Protein (AHRD V1)	0
138	Solyc09g064660	Small nuclear ribonucleoprotein Sm D1 (AHRD V1 ***- B6TXH2_MAIZE); contains Interpro domain(s) IPI	88.635
139	Solyc09g064670	LRR receptor-like serine/threonine-protein kinase, RLP	0.035
140	Solyc09g064680	Nbs-lrr, resistance protein	0.405
141	Solyc09g064690	Cc-nbs, resistance protein fragment	0
142	Solyc09g064710	Unknown Protein (AHRD V1)	0
143	Solyc09g064720	Dienelactone hydrolase family protein (AHRD V1 **-- D7L3C7_ARALY); contains Interpro domain(s) IPR0	24.64
144	Solyc09g064730	Heavy metal-associated domain containing protein expressed (AHRD V1 *-*- Q2QXS5_ORYSJ); contains Int	0.075
145	Solyc09g064740	RNA exonuclease 4 (AHRD V1 ***- B6T4V3_MAIZE); contains Interpro domain(s) IPR006055 Exonuclea	2.835
146	Solyc09g064750	Unknown Protein (AHRD V1)	0.9
147	Solyc09g064760	Unknown Protein (AHRD V1)	0
148	Solyc09g064770	F-box-like/WD repeat-containing protein TBL1XR1 (AHRD V1 ***- TBL1R_MOUSE); contains Interpro do	0
149	Solyc09g064780	At2g23590-like protein (Fragment) (AHRD V1 *-*- B2CXK6_CARAS)	5.585
150	Solyc09g064790	PAC (Fragment) (AHRD V1 **-- Q7DLY1_ARATH)	9.48
151	Solyc09g064800	Glycogen debranching enzyme (AHRD V1 ***- B1R1G0_CLOBU); contains Interpro domain(s) IPR006589	4.445
152	Solyc09g064810	Uncharacterized membrane protein C24H6.13 (AHRD V1 **-- YA7D_SCHPO); contains Interpro domain(s)	0.25
153	Solyc09g064820	Circadian clock coupling factor ZGT (AHRD V1 ***- Q94FM9_TOBAC)	0.19
154	Solyc09g064830	Unknown Protein (AHRD V1)	0
155	Solyc09g064840	Hepatoma-derived growth factor-related protein 3 (AHRD V1 *--- C1BWG5_ESOLU); contains Interpro dom	15.625
156	Solyc09g064850	Glutathione peroxidase (AHRD V1 ***- B9T4A6_RICCO); contains Interpro domain(s) IPR000889 Glutath	16.465
157	Solyc09g064860	CUE domain containing protein expressed (AHRD V1 *-*- Q7XG35_ORYSJ); contains Interpro domain(s) I	43.965
158	Solyc09g064870	Cbs domain containing protein expressed (Fragment) (AHRD V1 *--- A6N095_ORYSI); contains Interpro do	13.11
159	Solyc09g064880	ARGONAUTE 1 (AHRD V1 ***- D6RUV9_TOBAC)	0.58
160	Solyc09g064890	Unknown Protein (AHRD V1); contains Interpro domain(s) IPR001578 Peptidase C12, ubiquitin carboxyl-te	25.305
161	Solyc09g064900	AAA ATPase containing von Willebrand factor type A (AHRD V1 *--- B6THL9_MAIZE)	2.955
162	Solyc09g064910	tRNA dimethylallyltransferase (AHRD V1 *-*- D7WPA4_9BACI); contains Interpro domain(s) IPR002627 t	2.495
163	Solyc09g064920	BHLH transcription factor (AHRD V1 *-*- B6U846_MAIZE); contains Interpro domain(s) IPR011598 Heli	2.47
164	Solyc09g064930	R3H domain-containing protein C19orf22 homolog (AHRD V1 ***- CS022_BOVIN); contains Interpro doma	75.87
165	Solyc09g064940	Phenazine biosynthesis protein PhzF family (AHRD V1 ***- B4VKV9_9CYAN); contains Interpro domain(s)	103.255
166	Solyc09g064950	Leucine-rich repeat receptor-like protein kinase (AHRD V1 ***- Q9SN80_ARATH)	0.265
167	Solyc09g064960	LRR receptor-like serine/threonine-protein kinase, RLP	0
168	Solyc09g064970	F-box protein interaction domain containing protein (AHRD V1 ***- Q60D10_SOLDE); contains Interpro do	0
169	Solyc09g064980	LRR receptor-like serine/threonine-protein kinase, RLP	0
170	Solyc09g064990	LRR receptor-like serine/threonine-protein kinase, RLP	0
171	Solyc09g065000	Xylanase inhibitor (Fragment) (AHRD V1 **-- Q53IQ4_WHEAT); contains Interpro domain(s) IPR001461	2.51
172	Solyc09g065010	mRNA-capping enzyme subunit alpha (AHRD V1 *-** C4Y3U6_CLAL4); contains Interpro domain(s) IPR0	6.385
173	Solyc09g065020	F-box family protein (AHRD V1 ***- B9MYU0_POPTR)	3.98
174	Solyc09g065030	Os06g0207500 protein (Fragment) (AHRD V1 ***- Q0DDQ9_ORYSJ); contains Interpro domain(s) IPR004	12.705
175	Solyc09g065040	Embryo-abundant protein (Fragment) (AHRD V1 **-- Q3I3X0_PICAB); contains Interpro domain(s) IPR002	0.74
176	Solyc09g065050	Unknown Protein (AHRD V1)	0
177	Solyc09g065060	Mutator-like transposase (AHRD V1 *-*- Q9SL18_ARATH)	0
178	Solyc09g065070	Aluminum-activated malate transporter-like (AHRD V1 **-* Q6EPG5_ORYSJ); contains Interpro domain(s)	0.435
179	Solyc09g065080	RNA-binding protein (AHRD V1 *--* Q16HY2_AEDAE); contains Interpro domain(s) IPR000504 RNA rec	1.13
180	Solyc09g065090	Unknown Protein (AHRD V1)	0.08
181	Solyc09g065100	Transcription factor (AHRD V1 *--* Q9M4A8_MAIZE); contains Interpro domain(s) IPR001092 Basic heli	0.025
182	Solyc09g065110	Unknown Protein (AHRD V1)	6.9
183	Solyc09g065120	Preprotein translocase secY subunit (AHRD V1 ***- B6U7Q0_MAIZE); contains Interpro domain(s) IPR002	13.73
184	Solyc09g065130	Mitochondrial Rho GTPase 1 (AHRD V1 **** B2WA10_PYRTR); contains Interpro domain(s) IPR013567	17.735
185	Solyc09g065140	Unknown Protein (AHRD V1)	0.07
186	Solyc09g065150	Unknown Protein (AHRD V1)	0
187	Solyc09g065160	Dynamin family protein (AHRD V1 *--- D0MDN4_RHOM4); contains Interpro domain(s) IPR001401 Dyna	2.01
188	Solyc09g065170	Unknown Protein (AHRD V1)	0
189	Solyc09g065180	NAD-dependent epimerase/dehydratase (AHRD V1 **-- B7K7X4_CYAP7); contains Interpro domain(s) IPR	0.325
190	Solyc09g065190	Ran GTPase-activating protein 1 (AHRD V1 **-* A7M8K3_SOLTU); contains Interpro domain(s) IPR0035	15.915
191	Solyc09g065200	Cyclin-dependent kinase (AHRD V1 **** A6YTD1_CUCME); contains Interpro domain(s) IPR012389 Neg	32.675
192	Solyc09g065210	Kelch repeat and BTB domain-containing protein 4 (AHRD V1 *--- KBTB4_MOUSE); contains Interpro dom	25.88
193	Solyc09g065220	Unknown Protein (AHRD V1)	0
194	Solyc09g065230	Copalyl diphosphate synthase (AHRD V1 ***- Q9ST35_SOLLC); contains Interpro domain(s) IPR001906 T	0
195	Solyc09g065240	Patatin-like protein 3 (AHRD V1 ***- B6TPQ5_MAIZE); contains Interpro domain(s) IPR002641 Patatin	17.495
196	Solyc09g065250	Blue copper protein (AHRD V1 *--- B6TCC4_MAIZE); contains Interpro domain(s) IPR003245 Plastocyan	0
197	Solyc09g065260	Blue copper protein (AHRD V1 **-- B6U7P6_MAIZE); contains Interpro domain(s) IPR003245 Plastocyani	0
