# Supplementary material for: Nitric Oxide Overproduction in Tomato shr Mutant Shifts Metabolic Profiles and Suppresses Fruit Growth and Ripening
Source: Front Plant Sci. 2016 Nov 28;7:1714. doi: 10.3389/fpls.2016.01714 (PMC5124567; doi:10.3389/fpls.2016.01714)
Supplement: Supplementary Table S9 — Carotenoids content in fruits of WT and shr mutant at mature green (MG), breaker (BR) and red ripe stage (RR) stages of ripening. [file Table9.DOCX]

**Supplementary Material**

**Nitric oxide overproduction in tomato shr mutant alters cellular homeostasis and suppresses fruit growth and ripening**

*Reddaiah Bodanapu, Suresh Kumar Gupta, Pinjari Osman Basha, Kannabiran Sakthivel, Sadhna, Yellamaraju Sreelakshmi and Rameshwar Sharma*

**Corresponding author:** rameshwar.sharma@gmail.com

**Table S9**: Carotenoids content in fruits of WT and *shr* mutant at mature green (MG), breaker (BR) and red ripe stage (RR) stages of ripening.

| **Carotenoid** | **MG**  **μg/gm FW** | | **BR**  **μg/gm FW** | | **RR**  **μg/gm FW** | |
| --- | --- | --- | --- | --- | --- | --- |
|  | **WT** | ***shr*** | **WT** | ***shr*** | **WT** | ***shr*** |
| Phytoene | - | - | - | - | 13.46±2.10 | 18.43±1.52 ***** |
| Phytofluene | - | - | - | - | 3.4±0.43 | 4.80±0.45 ***** |
| Lycopene | 0.33±0.00 | 0.51±0.08 | 0.98±.34 | 0.63±0.13 | 45.24±6.59 | 43.34±2.75 |
| β-carotene | 2.09±0.36 | 3.38±0.23 | 4.89±0.41 | 2.81±0.57 | 4.73±1.12 | 5.16±0.18 |
| α-carotene | 0.31±0.01 | - | 0.31±0.01 | - | - | - |
| γ-carotene | - | - | 0.48±0.09 | 0.39±0.21 | 1.95±0.18 | 1.76±0.04 |
| Neoxanthin | 0.45±0.10 | 0.45±0.24 | 0.40±0.12 | 0.45±0.08 | - | - |
| Violaxanthin | 1.16±0.21 | 1.63±0.11 | 1.22±0.12 | 0.92±0.17 | 1.03±0.26 | 0.97±0.18 |
| Lutein | 2.52±0.58 | 3.51±0.38 | 2.79±0.27 | 2.07±0.42 | 2.55±0.37 | 2.41±0.04 |

The values are the mean ±SE obtained from five independent fruits of WT and *shr* mutant at each ripening stage. *indicates the significant difference between WT and *shr* at respective ripening stages tested by Student’s t test (P<0.05). - Sign indicates that no compound was detected in genotype or ripening stages
