# Supplementary material for: Nitric Oxide Overproduction in Tomato shr Mutant Shifts Metabolic Profiles and Suppresses Fruit Growth and Ripening
Source: Front Plant Sci. 2016 Nov 28;7:1714. doi: 10.3389/fpls.2016.01714 (PMC5124567; doi:10.3389/fpls.2016.01714)
Supplement: Supplementary Table S10 — List of metabolites identified in MG, BR, and RR stage of WT and shr fruits by GC-MS. [file Table10.DOCX]

**Supplementary Table S10 List of metabolites identified in MG, BR, and RR stage of WT and *shr* fruits by GC-MS.**

| **S. No** | **Compound name** | **Mature Green** | | **Log2Fold change** | **Breaker** | | **Log2Fold change** | **Red Ripe** | | **Log2Fold change** |
| --- | --- | --- | --- | --- | --- | --- | --- | --- | --- | --- |
|  |  | **WT** | ***shr*** |  | **WT** | ***shr*** |  | **WT** | ***shr*** |  |
| **Organic acids** | | | | | | | | | | |
| 1 | Lactate | 0.000±0.000 | 0.505±0.072 | 12.042** | 0.000±0.000 | 0.501±0.007 | 12.052** | 0.477±0.100 | 0.524±0.062 | 0.1365 |
| 2 | Acetate | 0.660±0.074 | 0.262±0.0124 | -1.333** | 0.452±0.041 | 0.166±0.021 | -1.440** | 0.281±0.021 | 0.146±0.025 | -0.9448* |
| 3 | Pyruvate | 1.147±0.196 | 1.096±0.068 | -0.0658 | 1.460±0.018 | 0.980±0.023 | -0.5748* | 1.188±0.158 | 0.903±0.142 | -0.3946 |
| 4 | Oxalate | 0.350±0.024 | 0.488±0.065 | 0.4814* | 0.770±0.186 | 0.570±0.020 | .-0.4329* | 0.490±0.118 | 0.489±0.044 | -0.0052 |
| 5 | Benzoate | 1.206±0.407 | 0.083±0.003 | -3.868* | 0.338±0.067 | 0.057±0.003 | -2.5764* | 0.124±0.020 | 0.049±0.005 | -1.3511* |
| 6 | Phosphate | 1.766±0.265 | 1.998±0.180 | 0.1781 | 2.385±0.561 | 1.510±0.112 | -0.6590 | 2.449±0.301 | 1.844±0.022 | -0.4094* |
| 7 | Nicotinate | 0.025±0.003 | 0.046±0.003 | 0.8692* | 0.048±0.006 | 0.080±0.007 | 0.7500* | 0.054±0.008 | 0.083±0.011 | 0.6277* |
| 8 | Succinate | 3.035±0.096 | 0.715±0.008 | -2.086** | 1.704±0.066 | 0.552±0.035 | -1.624** | 1.076±0.013 | 0.614±0.056 | -0.808** |
| 9 | Methylsucciniate | 0.874±0.045 | 0.218±0.035 | -2.006** | 0.566±0.012 | 0.167±0.007 | -1.760** | 0.232±0.005 | 0.116±0.016 | -1.002** |
| 10 | Fumarate | 4.367±1.344 | 5.200±0.606 | 0.2591 | 8.823±1.029 | 5.348±0.222 | -0.7223** | 5.622±0.153 | 4.848±0.400 | -0.2138 |
| 11 | Glutarate | 0.746±0.044 | 0.103±0.003 | -2.8526** | 0.355±0.026 | 0.075±0.005 | -2.2380** | 0.139±0.014 | 0.087±0.001 | -0.6664* |
| 12 | Hydrocinnamate | 0.013±0.004 | 0.000±0.000 | -6.288** | 0.006±0.002 | 0.002±0.002 | -1.700 | 0.002±0.000 | 0.002±0.001 | -0.01 |
| 13 | Malate | 4.575±1.025 | 7.697±2.040 | 0.7505 | 10.256±1.90 | 12.80±0.773 | 0.3205 | 2.028±0.424 | 5.884±0.636 | 1.536* |
| 14 | Tartrate | 0.000±0.000 | 0.010±0.005 | 6.1918** | 0.000±0.000 | 0.006±0.000 | 5.5359** | 0.021±0.000 | 0.039±0.009 | 0.923* |
| 15 | cis-Aconitate | 0.013±0.003 | 0.231±0.083 | 4.105* | 0.064±0.029 | 0.406±0.006 | 2.674** | 0.166±0.031 | 0.413±0.108 | 1.316* |
| 16 | Isocitrate | 0.004±0.001 | 0.010±0.005 | 1.396 | 0.007±0.000 | 0.015±0.000 | 1.103* | 0.003±0.001 | 0.013±0.000 | 1.984* |
| 17 | Citrate | 0.232±0.027 | 2.611±0.381 | 3.4933* | 2.038±0.566 | 6.563±0.206 | 1.6872** | 2.648±0.704 | 7.538±1.24 | 1.509** |
| 18 | Dehydroascorbate | 0.007±0.003 | 0.024±0.007 | 1.7800* | 0.014±0.001 | 0.042±0.001 | 1.5560* | 0.030±0.004 | 0.084±0.013 | 1.500** |
| 19 | Quinate | 0.176±0.030 | 0.156±0.009 | -0.1770 | 0.190±0.009 | 0.156±0.045 | -0.2818 | 0.307±0.012 | 0.308±0.074 | 0.0073 |
| 20 | trans-Caffeate | 0.032±0.018 | 0.081±0.006 | 1.342* | 0.077±0.010 | 0.116±0.019 | 0.5880* | 0.055±0.016 | 0.131±0.022 | 1.243** |
| **Sugars** | | | | | | | | | | |
| 21 | Fructose | 44.386±0.296 | 48.322±0.403 | 0.1225** | 45.595±0.173 | 49.451±0.388 | .1171** | 47.31±0.654 | 49.645±0.195 | 0.0694** |
| 22 | Tagatose | 26.259±0.099 | 27.268±0.165 | 0.0543** | 26.247±0.145 | 28.561±0.345 | 0.1218** | 26.60±0.248 | 28.654±0.360 | 0.107** |
| 23 | Threonic acid-1,4-lactone | 0.021±0.000 | 0.032±0.012 | 0.6190 | 0.028±0.001 | 0.027±0.006 | 0.00141 | 0.029±0.014 | 0.027±0.011 | -0.1513 |
| 24 | Erythrono-1,4-lactone | 0.008±0.002 | 0.009±0.000 | 0.1766 | 0.008±0.001 | 0.008±0.000 | 0.0895 | 0.010±0.000 | 0.009±0.0021 | -0.1255 |
| 25 | Threonate | 0.120±0.010 | 0.044±0.005 | -1.442** | 0.061±0.007 | 0.027±0.010 | -1.1666* | 0.061±0.005 | 0.024±0.009 | -1.350** |
| 26 | Erythronate | 0.044±0.006 | 0.063±0.002 | 0.5266* | 0.030±0.006 | 0.064±0.017 | 1.123 | 0.057±0.012 | 0.045±0.012 | -0.3312 |
| 27 | Arabinopyranose | 0.057±0.005 | 0.027±0.002 | -1.1147** | 0.040±0.003 | 0.029±0.003 | -0.4550* | 0.052±0.003 | 0.028±0.004 | -0.894** |
| 28 | Xylose | 0.069±0.003 | 0.060±0.008 | -0.2171 | 0.066±0.000 | 0.091±0.008 | 0.4711* | 0.104±0.001 | 0.089±0.000 | -0.2174* |
| 29 | Arabinose | 0.175±0.003 | 0.202±0.031 | 0.2122 | 0.202±0.005 | 0.296±0.058 | 0.5489* | 0.380±0.013 | 0.352±0.026 | -0.1108 |
| 30 | Ribose | 0.055±0.006 | 0.054±0.005 | -0.0304 | 0.075±0.001 | 0.070±0.008 | -0.0832 | 0.158±0.027 | 0.093±0.010 | -0.769* |
| 31 | Ribofuranose | 0.160±0.013 | 0.059±0.005 | -1.437** | 0.104±0.003 | 0.061±0.007 | -0.7856** | 0.123±0.076 | 0.054±0.006 | -1.181** |
| 32 | Rhamnose | 0.047±0.000 | 0.044±0.006 | -0.100 | 0.041±0.002 | 0.050±0.002 | 0.2930* | 0.063±0.005 | 0.056±0.008 | -0.1631 |
| 33 | Fructofuranose | 0.051±0.013 | 0.035±0.006 | -0.5631 | 0.028±0.010 | 0.036±0.008 | 0.3981 | 0.019±0.007 | 0.189±0.033 | 3.331** |
| 34 | Fructopyranose | 0.619±0.186 | 0.292±0.029 | -1.0834* | 0.324±0.014 | 0.360±0.032 | 0.1516 | 0.274±0.032 | 0.368±0.026 | 0.4288* |
| 35 | Glucuronic acid γ-lactone | 0.000±0.000 | 0.000±0.000 | -0.5383 | 0.005±0.003 | 0.006±0.000 | 0.2335 | 0.011±0.002 | 0.013±0.001 | 0.2127* |
| 36 | Glucopyranose | 0.542±0.063 | 0.318±0.007 | -0.7683* | 0.000±0.000 | 0.582±0.256 | 12.22** | 0.000±0.000 | 0.958±0.143 | 13.10** |
| 37 | Glucose | 27.206±2.29 | 22.763±0.591 | -0.2572 | 27.456±1.32 | 24.484±0.358 | -0.1652* | 22.95±1.165 | 19.408±2.00 | -0.2423 |
| 38 | Mannose | 23.156±1.11 | 19.463±1.957 | -0.2572 | 22.007±2.059 | 18.725±0.070 | -0.2329 | 19.41±0.829 | 15.949±1.60 | -0.2839 |
| 39 | Galactose | 28.106±1.48 | 20.207±0.691 | -0.4760* | 25.072±2.458 | 23.573±0.300 | -0.0889 | 24.12±1.159 | 20.229±2.28 | -0.25411 |
| 40 | Glucuronate | 0.139±0.014 | 0.164±0.045 | 0.2408 | 0.209±0.012 | 0.211±0.033 | 0.0135 | 1.330±0.139 | 1.066±0.098 | -03195* |
| 41 | Galacturonate | 0.000±0.000 | 0.000±0.000 | -0.2580 | 0.000±0.000 | 0.000±0.000 | -0.1883 | 0.182±0.023 | 0.135±0.016 | -0.4326** |
| 42 | Gluconate | 0.195±0.026 | 0.131±0.024 | -0.579 | 0.217±0.015 | 0.241±0.009 | 0.1548* | 0.385±0.048 | 0.264±0.061 | -0.5439* |
| 43 | Glucarate | 0.188±0.027 | 0.107±0.044 | -0.8169 | 0.154±0.018 | 0.106±0.008 | -0.5307 | 0.213±0.052 | 0.106±0.020 | -1.008* |
| 44 | Galactarate | 0.002±0.001 | 0.003±0.001 | 0.5046 | 0.007±0.001 | 0.007±0.000 | -0.1373 | 0.020±0.005 | 0.019±0.002 | -0.1156 |
| 45 | myo-Inositol | 6.548±0.925 | 6.640±1.499 | 0.0202 | 6.628±0.960 | 5.780±0.561 | -0.1974 | 5.693±0.942 | 5.727±1.403 | 0.0086 |
| 46 | Sucrose | 0.348±0.935 | 0.452±0.213 | 0.0380 | 0.474±0.084 | 0.692±0.172 | 0.54851 | 0.375±0.045 | 0.347±0.057 | -0.112 |
| 47 | Galactonate | 0.156±0.029 | 0.137±0.021 | -0.1915 | 0.192±0.022 | 0.286±0.081 | 0.5699* | 0.380±0.041 | 0.309±0.022 | -0.3005 |
| **Amino acids and Amines** | | | | | | | | | | |
| 48 | I-Alanine | 1.200±0.240 | 1.085±0.136 | -0.1448 | 1.025±0.051 | 0.674±0.239 | -0.6058 | 1.462±0.308 | 0.954±0.209 | -0.6157 |
| 49 | Isoleucine | 0.023±0.005 | 0.147±0.048 | 2.7017** | 0.130±0.022 | 0.063±0.002 | -1.043* | 0.024±0.001 | 0.105±0.024 | 2.1257* |
| 50 | Valine | 0.757±0.066 | 0.687±0.022 | -0.1406 | 0.688±0.206 | 0.475±0.176 | -0.5338 | 0.188±0.032 | 0.093±0.034 | -1.023* |
| 51 | Leucine | 0.165±0.035 | 0.196±0.025 | 0.2453 | 0.254±0.042 | 0.222±0.074 | -0.1938 | 0.133±0.025 | 0.116±0.009 | -0.2008 |
| 52 | Glycine | 0.302±0.045 | 0.373±0.006 | 0.3037 | 0.482±0.105 | 0.162±0.033 | -1.574* | 0.222±0.033 | 0.204±0.029 | -0.1237 |
| 53 | Serine | 1.085±0.461 | 1.013±0.209 | -0.0991 | 1.442±0.181 | 1.022±0.233 | -0.4959* | 0.496±0.144 | 0.555±0.158 | 0.1606 |
| 54 | Threonine | 0.381±0.022 | 0.469±0.064 | 0.2991 | 0.502±0.135 | 0.526±0.174 | 0.0671 | 0.213±0.064 | 0.242±0.048 | 0.1877 |
| 55 | beta-Alanine | 0.048±0.031 | 0.071±0.035 | 0.5601 | 0.124±0.009 | 0.061±0.005 | -1.013** | 0.092±0.010 | 0.173±0.073 | 0.909* |
| 56 | Aspartate | 0.142±0.039 | 0.142±0.019 | 0.0028 | 0.275±0.077 | 0.071±0.006 | -1.948** | 0.078±0.008 | 0.044±0.010 | -0.833* |
| 57 | Proline, 5-oxo | 3.807±1.773 | 6.720±0.878 | 0.8199 | 11.500±1.95 | 10.38±2.646 | -0.1453 | 8.099±1.522 | 8.879±2.371 | 0.1325 |
| 58 | GABA | 6.412±0.722 | 5.069±1.087 | -0.3392 | 8.974±2.840 | 7.383±2.73 | -0.2815 | 5.723±0.845 | 2.712±0.455 | -1.078* |
| 59 | Cysteine | 0.031±0.004 | 0.045±0.001 | 0.5419** | 0.052±0.000 | 0.047±0.001 | -0.1288 | 0.055±0.054 | 0.079±0.054 | 0.531** |
| 60 | Glutamate | 0.409±0.068 | 0.195±0.037 | -1.065** | 0.302±0.031 | 0.218±0.035 | -0.4673* | 0.392±0.043 | 0.258±0.074 | -0.6023* |
| 61 | Phenylalanine | 0.117±0.032 | 0.160±0.057 | 0.4515 | 0.177±0.070 | 0.232±0.062 | 0.3907 | 0.090±0.023 | 0.119±0.014 | 0.4072 |
| 62 | Asparagine | 0.001±0.000 | 0.018±0.005 | 3.633* | 0.005±0.003 | 0.039±0.014 | 3.052* | 0.003±0.000 | 0.026±0.014 | 3.234* |
| 63 | Glutamine | 0.008±0.003 | 0.064±0.019 | 3.028* | 0.020±0.01 | 0.471±0.058 | 4.5239** | 0.056±0.059 | 0.126±0.010 | 1.166 |
| 64 | Tyrosine | 0.00±0.000 | 0.484±0.087 | 11.55** | 0.00±0.00 | 0.35±0.044 | 11.31** | 0.00±0.00 | 0.136±0.0153 | 10.31** |
| 65 | Tryptophan | 0.015±0.000 | 0.026±0.001 | 0.7718** | 0.023±0.001 | 0.039±0.001 | 0.7330** | 0.024±0.002 | 0.029±0.000 | 0.2783* |
| 66 | Alanine, 3-cyano- | 0.016±0.007 | 0.183±0.059 | 3.481* | 0.150±0.043 | 0.259±0.043 | 0.7911* | 0.046±0.021 | 0.116±0.028 | 1.3429* |
| 67 | Putrescine | 0.053±0.008 | 0.114±0.021 | 1.1086* | 0.183±0.034 | 0.213±0.040 | 0.2210 | 0.047±0.013 | 0.064±0.0103 | 0.4586 |
| 68 | Ornithine | 0.040±0.019 | 0.071±0.010 | 0.828 | 0.089±0.008 | 0.098±0.042 | 0.1363 | 0.044±0.021 | 0.079±0.0151 | 0.8331 |
| 69 | Ornithine-1,5-lactam | 0.041±0.029 | 0.434±0.051 | 3.3978** | 0.356±0.036 | 1.447±0.399 | 2.0245* | 0.063±0.024 | 0.384±0.06 | 2.603** |
| 70 | Ethanolamine | 0.055±0.007 | 0.165±0.074 | 1.580 | 0.079±0.029 | 0.089±0.023 | 0.180 | 0.056±0.021 | 0.070±0.007 | 0.313 |
| 71 | Aminomethane | 0.005±0.002 | 0.011±0.007 | 1.321 | 0.004±0.001 | 0.004±0.001 | -0.052 | 0.004±0.001 | 0.002±0.000 | -0.795 |
| 72 | Hydroxylamine | 0.018±0.003 | 0.135±0.008 | 2.907** | 0.027±0.002 | 0.137±0.006 | 2.333** | 0.122±0.010 | 0.169±0.026 | 0.470* |
| **Fatty acids** | | | | | | | | | | |
| 73 | Caproate | 0.034±0.009 | 0.011±0.003 | -1.561** | 0.018±0.001 | 0.007±0.000 | -1.192** | 0.010±0.002 | 0.008±0.000 | -0.464 |
| 74 | Heptanoate | 0.022±0.006 | 0.004±0.000 | -2.511** | 0.008±0.001 | 0.004±0.000 | -0.983** | 0.005±0.000 | 0.004±0.000 | -0.037 |
| 75 | Octanoate | 0.115±0.047 | 0.057±0.009 | -1.019* | 0.097±0.007 | 0.045±0.008 | -1.081** | 0.037±0.014 | 0.018±0.006 | -1.024 |
| 76 | Nonanoate | 0.064±0.026 | 0.015±0.002 | -2.092* | 0.026±0.001 | 0.014±0.002 | -0.857* | 0.016±0.003 | 0.014±0.002 | -0.223 |
| 77 | Decanoate | 0.044±0.013 | 0.008±0.000 | -2.528** | 0.013±0.000 | 0.007±0.001 | -0.937** | 0.008±0.000 | 0.008±0.000 | 0.062 |
| 78 | Undecanoate | 0.026±0.006 | 0.000±0.001 | -8.13** | 0.015±0.001 | 0.000±0.000 | -6.99** | 0.008±0.000 | 0.006±0.001 | -0.4882 |
| 79 | Laurate | 0.138±0.040 | 0.021±0.009 | -2.749** | 0.054±0.004 | 0.020±0.001 | -1.407** | 0.025±0.002 | 0.021±0.000 | -0.234 |
| 80 | Myristate | 0.096±0.014 | 0.025±0.003 | -1.961** | 0.048±0.011 | 0.029±0.002 | -0.729* | 0.036±0.004 | 0.000±0.000 | -8.682** |
| 81 | Palmitolate | 0.077±0.012 | 0.017±0.001 | -2.166** | 0.029±0.003 | 0.021±0.003 | -0.469* | 0.023±0.005 | 0.034±0.008 | 0.5303 |
| 82 | Palmitate | 7.760±1.56 | 2.590±0.266 | -1.583** | 3.009±0.260 | 3.020±0.318 | 0.005 | 2.860±0.194 | 3.332±0.585 | 0.220 |
| 83 | Margarate | 0.193±0.01 | 0.102±0.011 | -0.916** | 0.106±0.006 | 0.139±0.011 | 0.386* | 0.110±0.018 | 0.143±0.025 | 0.388 |
| 84 | Linolate | 0.056±0.017 | 0.040±0.003 | -0.490 | 0.038±0.001 | 0.045±0.003 | 0.268* | 0.025±0.007 | 0.048±0.021 | 0.990 |
| 85 | Octadecenoate, 6-(Z) | 0.110±0.002 | 0.029±0.003 | -1.932** | 0.024±0.003 | 0.037±0.001 | 0.624* | 0.022±0.002 | 0.038±0.011 | 0.829 |
| 86 | Oleate | 0.119±0.002 | 0.040±0.005 | -1.583** | 0.079±0.012 | 0.045±0.003 | -0.821** | 0.039±0.003 | 0.053±0.015 | 0.433 |
| 87 | Stearate | 8.177±0.620 | 3.799±0.340 | -1.105** | 4.343±0.361 | 4.611±0.327 | 0.086 | 4.197±0.749 | 5.093±1.118 | 0.279 |
| 88 | Arachidate | 0.146±0.036 | 0.085±0.007 | -0.784* | 0.156±0.021 | 0.089±0.002 | -0.805* | 0.088±0.003 | 0.103±0.016 | 0.228 |
| 89 | Docosanoate | 0.041±0.019 | 0.072±0.007 | 0.806* | 0.109±0.017 | 0.075±0.001 | -0.534* | 0.077±0.000 | 0.075±0.003 | -0.032 |
| **Others** | | | | | | | | | | |
| 90 | Velarate | 0.027±0.007 | 0.039±0.007 | 0.564 | 0.043±0.001 | 0.042±0.006 | -0.0356 | 0.049±0.007 | 0.042±0.011 | -0.2434 |
| 91 | Dehydroabietate | 0.035±0.000 | 0.025±0.000 | -0.4701** | 0.021±0.000 | 0.023±0.001 | 0.1164 | 0.020±0.001 | 0.042±0.003 | 1.088** |
| 92 | Tryptamine, 5-hydroxy | 0.022±0.005 | 0.029±0.001 | 0.3533 | 0.046±0.004 | 0.044±0.005 | -0.0920 | 0.038±0.018 | 0.057±0.020 | 0.0601 |
| 93 | 2-Furancarboxylate | 0.314±0.145 | 0.034±0.001 | -3.189** | 0.072±0.007 | 0.024±0.002 | -1.6021** | 0.043±0.004 | 0.030±0.001 | -0.5323** |
| 94 | 2-Hydroxyvalerate | 0.026±0.002 | 0.005±0.000 | -2.3080** | 0.012±0.001 | 0.003±0.000 | -2.1829** | 0.007±0.001 | 0.005±0.001 | -0.5914 |
| **Neucleotides** | | | | | | | | | | |
| 95 | Adenosine | 0.000±0.000 | 0.000±0.000 | -0.240 | 0.001±0.000 | 0.002±0.000 | 0.756** | 0.005±0.000 | 0.013±0.000 | 1.27** |
| 96 | Guanidine | 0.000±0.000 | 0.020±0.001 | 7.19** | 0.044±0.028 | 0.050±0.022 | 0.1715 | 0.008±0.001 | 0.025±0.006 | 1.586* |

The value represents the mean of ≥3-5± S. D. The star mark on log2 fold change value shows significant difference between WT and *shr* at specified ripening stage, * P≤ 0.05, **P≤ 0.001. The light grey color shows, difference between WT and *shr* is greater than log2 fold change 0.5 (equals to 1.5 fold obtained by dividing shr /WT) either in increase or decrease. MG- Mature green, BR- Breaker, RR- Red Ripe. All pairwise comparison was performed using the One way Annova (Student-Newman-Keuls Method, P≤0.05).
